# Supplementary material for: Strain regulates the photovoltaic performance of thick-film perovskites
Source: Nat Commun. 2024 Mar 22;15:2579. doi: 10.1038/s41467-024-47019-8 (PMC10960009; doi:10.1038/s41467-024-47019-8)
Supplement: Supplementary file 1 — Supplementary Information [file 41467_2024_47019_MOESM1_ESM.pdf]

Supplementary Materials for

Strain Regulates the Photovoltaic Performance of Thick-Film Perovskites

Pengju Shi[1,2†], Jiazhe Xu[1,2†], Ilhan Yavuz[3†], Tianyi Huang[4], Shaun Tan[4], Ke Zhao[1,2], Xu Zhang[1,2], Yuan Tian[1,2], Sisi Wang[2], Wei Fan[1,2], Yahui Li[1,2], Donger Jin[1], Xuemeng Yu[5], Chenyue Wang[6], Xingyu Gao[6], Zhong Chen[7], Enzheng Shi[1], Xihan Chen[5], Deren Yang[1], Jingjing Xue[1,8*], Yang Yang[4*] and Rui Wang[2*]

[1]State Key Laboratory of Silicon Materials and School of Materials Science and Engineering, Zhejiang University, Hangzhou 310027, China

[2]Research Center for Industries of the Future, School of Engineering, Westlake University and Institute of Advanced Technology, Westlake Institute for Advanced Study, Hangzhou 310024, China

[3]Department of Physics, Marmara University, Ziverbey, Istanbul 34722, Turkiye

[4]Department of Materials Science and Engineering and California NanoSystems Institute, University of California Los Angeles, CA 90095, USA

[5]Department of Mechanical and Energy Engineering, Southern University of Science and Technology, Shenzhen, Guangdong 518055, China

[6]Shanghai Synchrotron Radiation Facility (SSRF), Zhangjiang Lab, Shanghai Advanced Research Institute, Chinese Academy of Sciences, 239 Zhangheng Road, Shanghai 201204, China

[7]Instrumentation and Service Center for Molecular Sciences, Westlake University, 18 Shilongshan Road, Hangzhou 310024, Zhejiang Province, China

[8]Shangyu Institute of Semiconductor Materials, Shaoxing 312300, China

*Correspondence and requests for materials should be addressed to J.X. (email: jjxue@zju.edu.cn) or to Y.Y. (email: yangy@ucla.edu) or to R.W. (email: wangrui@westlake.edu.cn)

†These authors contributed equally to this work

Supplementary Note. 1

Kinetic models of carriers of perovskite

Transient reflection kinetics modelling

The measured surface carrier kinetics can be modelled with diffusion and surface recombination model, the detailed description of which can be found in previously published literature[1]. Since the TRS is done on films and there are no significant charge transfer happening within the few ns probe window, we can still assume the majority of the carriers within the interaction region follow one dimensional diffusion equation

$$\frac{\partial N(x,t)}{\partial t} = D\frac{\partial^2 N(x,t)}{\partial x^2} - \frac{N(x,t)}{\tau_B} \#(1)$$

where $N(x, t)$ is the carrier density as a function of depth (x) and time (t), D is the ambipolar diffusion coefficient (D_{ab}) and τ_B is the bulk carrier lifetime. Generally, in perovskite material, τ_B is on the order of hundreds of ns, in the 5 ns probe window, it can be neglected. The initial condition for equation (1) is then given by carrier generation profile

$N(x,0) = N_0 \cdot exp(-\alpha x) \#(2)$ere N_0 is the initial surface carrier density. If the traces are normalized, then N_0 is equal to 1. The values for the absorption coefficient at different pump-photon energies for both samples are known. The boundary condition can be described by the followings, for the front surface, surface recombination will dominate (S_F), and it is defined as

$$\frac{\partial N(x,t)}{\partial t}|_{x=0} = \frac{S_F}{D} N(0,t) \#(3)$$

For the back surface, we assume an equal surface recombination contribution

$$\frac{\partial N(x,t)}{\partial t}|_{x=L_{int}} = -\frac{S_{int}}{D} N(L_{int},t) \#(4)$$

In this model, L_{int} is the thickness of the film. S_F and S_{int} are kept the same since the front and back surface recombination are similar.

Supplementary Note. 2

Strain distribution in perovskite films via GIWAXS

Grazing-Incidence Wide-Angle X-ray Scattering (GIWAXS) was used to detect the spatial distribution of strain in perovskite films by tuning the incident angle (θ) from 0.3° to 1.0° with a step increase of 0.1° (Supplementary Fig. 27). The X-ray energy for the GIWAXS measurement is 9.9 KeV with the resolution of the GIWAXS experiments in this work is 0.0027 Å. The measured data was then fitted with single peak Gaussian function[2].

Supplementary Note. 3

Measurements of electron and hole mobility

In perovskite materials, the charge carriers are electron and holes. Space charge limit current (SCLC) was measured with the Keithley 2450 source measure unit under dark condition[3]. The current related the applied voltage were shown as follows. J is the current density, L is the film thickness, V the applied voltage, ε is vacuum dielectric constant, ε_0 is the dielectric constant of perovskite, μ is mobility.

$$J = \frac{9}{8}\varepsilon\varepsilon_0\mu\frac{V^2}{L^3}exp\left(\beta\sqrt{\frac{V}{L}}\right)\#(5)$$

$$\frac{JL^3}{V^2} = \frac{9}{8}\varepsilon\varepsilon_0\mu exp\left(\beta\sqrt{\frac{V}{L}}\right)\#(6)$$

$\ln\left(\frac{JL^3}{V^2}\right) = ln\left(\frac{9}{8}\varepsilon\varepsilon_0\mu\right) + \beta\sqrt{\frac{V}{L}}\#(7)$the intercept is $ln\left(\frac{9}{8}\varepsilon\varepsilon_0\mu\right)$, and then the mobility is：

$$\mu = \frac{8}{9\varepsilon\varepsilon_0}exp(A)\#(8)$$

$$\varepsilon\varepsilon_0 = 40 \times 10^{-14}\frac{F}{cm}\#(9)$$

Electron only mobility was detected with the structure of ITO/SnO_2/perovskite/PCBM/Au. Hole only mobility was detected with the structure of ITO/PEDIT:PSS/perovskite/ Spiro-OMeTAD/Au.

Supplementary Note. 4

Density Functional Theory Analysis

Computational methodology. Density Functional Theory (DFT) methodology is employed for theoretical calculations. A HSEsol[4, 5] type generalized gradient approximation for the exchange-correlation functional and dispersion correction to the electronic energy, based on Grimme's DFT-D3 scheme, were used[6, 7]. Plane-wave basis sets with 300 eV cut-off were used in geometry optimization calculations a further 400 eV cut-off were used for property predictions. Projector-augmented-wave (PAW) pseudopotentials were chosen to describe valence-core interactions. Atomic positions and supercell were both optimized using a conjugate gradient algorithm until all residual forces are smaller than 0.02 eV/Å. 4*4*4 Γ-center k-point grid was used for Brillouin-zone sampling. All first-principles calculations were carried out with the VASP code[8, 9]. For DFT calculations including strain, only lattice parameter in the c-direction were constrained, while others are allowed to relax. The bulk c-axis value is found to be 6.36 Å and our XRD data showed that this value becomes 6.321 Å and 6.294 Å for 2.0 M and 1.4 M perovskite, respetively. We use these lattice values accordingly in our 1.4 M and 2.0 M perovskite band-structure calculations including strain.

Effective-mass calculations. Hole and electron transport effective mass is calculated from the tensor relation

$$\left[m_{eff}\right]_{ij}^{-1} = \frac{1}{\hbar^2} \frac{\partial^2 E}{\partial k_i \partial k_i} \#(10)$$

where k_i is the reciprocal space vector and E is the band-resolved energy. A five-point numerical differentiation method and $S \rightarrow R \rightarrow U$ k-path (Supplementary Fig. 29), including the valence band maximum (VBM) and conduction band minimum (CBM), is used to predict hole and electron m_{eff}.

Supplementary Note. 5

Measurements of carrier concentration by CV

Carrier concentration was obtained by the capacitance-voltage (*C-V*) measurement via Cryogen-free cryogenic probe stations & 4200A-SCS (CRX-4K)[10, 11]. The carrier concentration was calculated according to the model of abrupt p-n junction. In the Figure 3e, the slope can be calculated in this figure and the Carrier concentration is gotten by:

$$slope = \frac{2}{\varepsilon\varepsilon_0 A^2 e N_D} \#(11)$$

where A is the effective area of the samples, e is the electron charge. The electron concentration is measured with the device structure of ITO/SnO_2/perovskite/PCBM/Au.

Supplementary Note. 6

Measurements of carrier concentration by DLCP

The DLCP measurement were conducted in the same DC bias range with that for the standard *C-V* measurement. The amplitude of the AC biases ranged from 20 to 140 mV. For each AC bias, an additional offset DC voltage was applied to keep the maximum forward bias constant. The measured capacitances at each DC bias were gathered and fitted with a polynomial function to obtain C_0 and C_1. The derivation of the carrier density is based on the nonlinear relationship between the change of charges (δQ) and the perturbation AC bias (δV)[12]:

$$\frac{\delta Q}{\delta V} = C_0 + C_0 \delta V + C_0 (\delta V)^2 + \cdots \#(12)$$

With the determination of C_0 and C_1, the carrier density (N) from the junction barrier is calculated by:

$$N = -\frac{C_0^3}{2q\varepsilon A^2 C_1} \#(13)$$

where q is the elementary charge, ε is the dielectric constant of the semiconductor and A is the active area of the junction.

Different from CV measurements that applies DC bias in the range of 0 to 1 V, DLCP detected the capacitance signal by applying AC bias under a changeable DC bias to maintain a constant maximum bias. Via the DLCP results, we extracted the electron concentration which indicated a similar trend as CV measurements, with the value nearly equal to that from CV plots.

Supplementary Note. 7

Calculation of trap Density of States (tDOS)

The trap density of states (tDOS) were extracted from CV and CF plots according to the equations[13]:

$$N_T(E_\omega) = -\frac{V_{bi}}{qW}\frac{dC}{d\omega}\frac{\omega}{k_B T} \#(14)$$

$$E_\omega = k_B T ln\left(\frac{\beta T^2}{\omega}\right) \#(15)$$

where V_{bi} is the built-in potential, W is the depletion width, q is the elementary charge, C is the capacitance, and ω is the applied angular frequency.

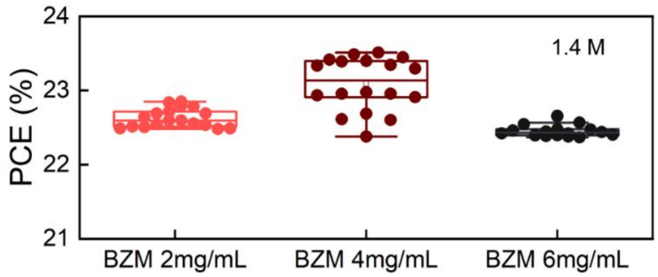

Supplementary Fig. 1 | Modifying BZM concentration in 1.4 M. PCE statistics by varying BZM concentrations in 1.4 M. Centre line, median; box limits, 25th and 75th percentiles; curve, normal distribution curve; whiskers, outliers.

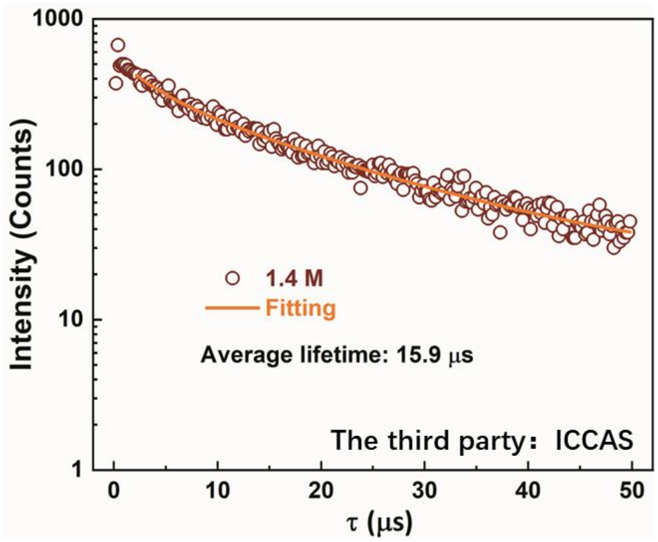

Supplementary Fig. 2 | A third party verification of the TRPL measurement of 1.4 M done by the Institute of Chemistry, Chinese Academy of Science (ICCAS). The sample was measured by a laser of 483.6 nm with an average power of 5 mW. The calculated lifetime by ICCAS was determined to be 15.9 μs.

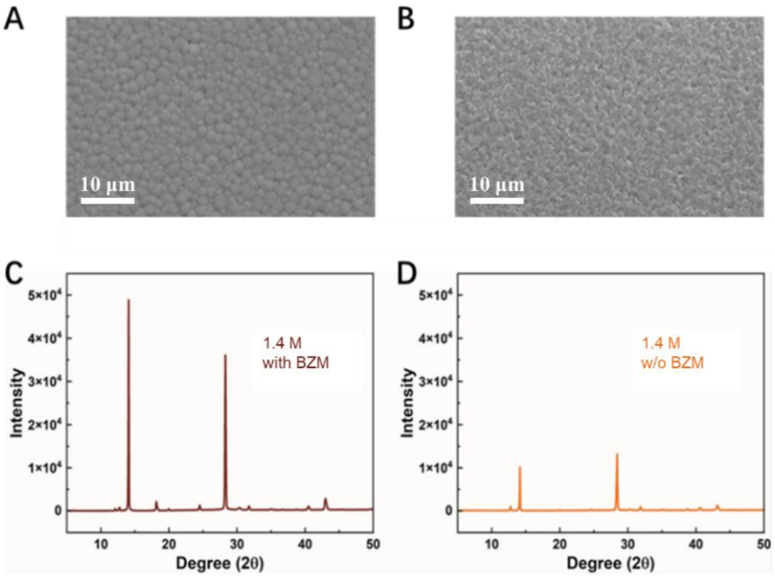

Supplementary Fig. 3 | Investigation on the crystallinity of 1.4 M perovskite films with and without BZM. Top views of scanning electron microscope of (a) the 1.4 M perovskite film with BZM and (b) the 1.4 M perovskite film without BZM. XRD patterns of (c) the 1.4 M perovskite film and (d) the 1.4 M perovskite film without BZM.

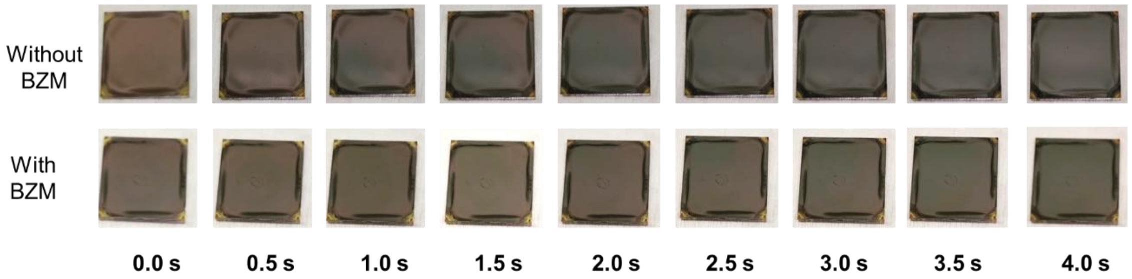

Supplementary Fig. 4 | Comparison of the phase transformation rates of 1.4 M perovskite films with and without BZM. Photos of the perovskite films with and without BZM during the annealing process.

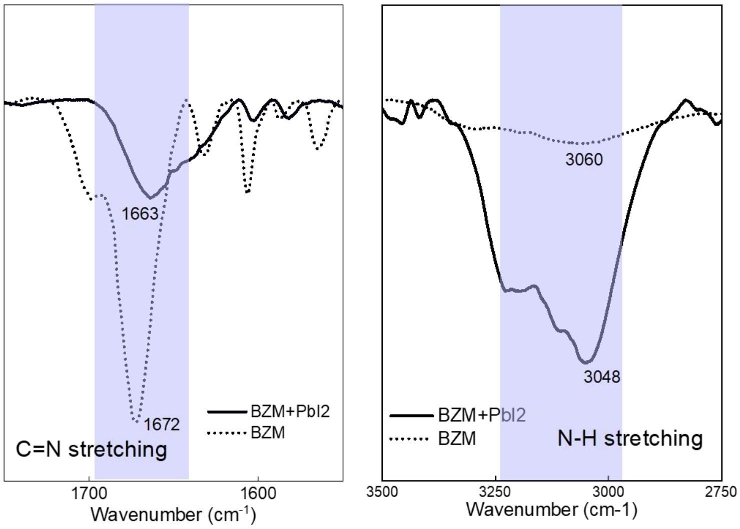

Supplementary Fig. 5 | Interactions between BZM and PbI_2. FTIR spectra of pure BZM and BZM mixed with PbI_2. The C=N stretching vibration, and the N-H stretching vibration of BZM shifted from 1672 to 1663 cm^{-1}, and from 3060 to 3048 cm^{-1} respectively, after mixing with PbI_2, suggesting the binding of BZM to the Pb^{2+} through the amidinium group.

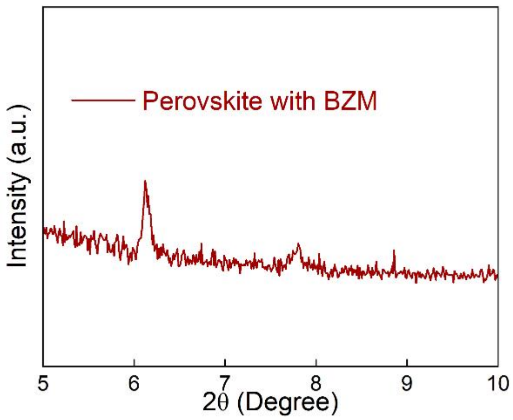

Supplementary Fig. 6 | Low-dimensional perovskite phases when introduced BZM. Enlarged XRD patterns at low diffraction angles of the 1.4 M perovskite film with BZM.

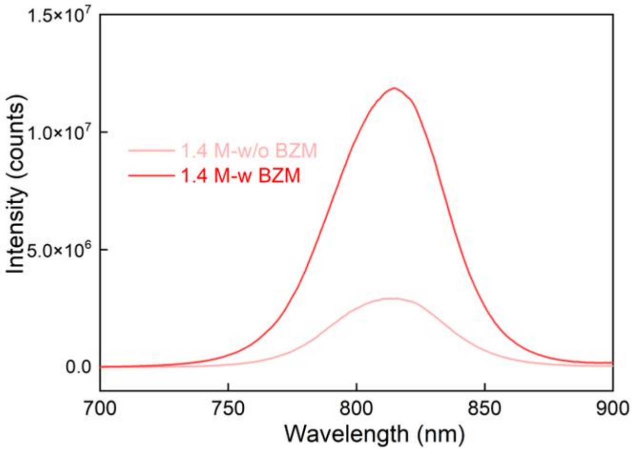

Supplementary Fig. 7 | Defects passivation when introduced BZM. PL spectra of the 1.4 M perovskite thin films with and without introducing BZM.

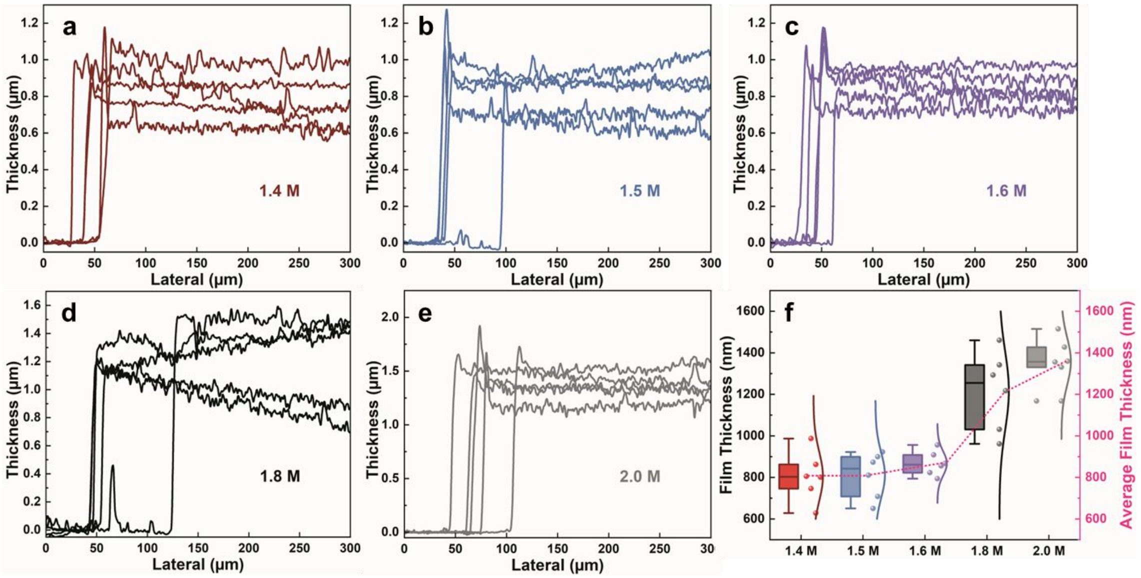

Supplementary Fig. 8 | Comparison of perovskite film thicknesses. Film thickness of (a) 1.4 M, (b) 1.5 M, (c) 1.6 M, (d) 1.8 M and (e) 2.0 M perovskite film measured by step profiler. (f) Statistics of the thickness in these five perovskite films with the average thickness shown by red dot line.

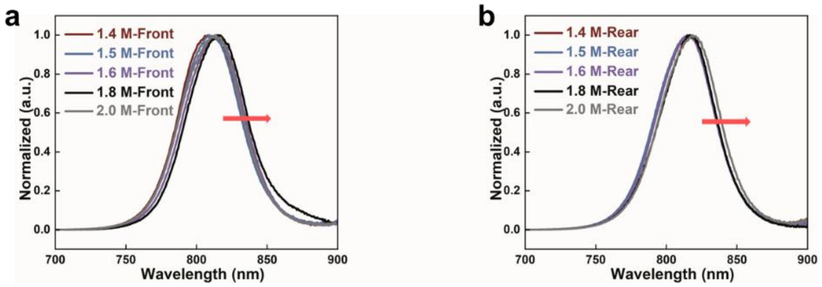

Supplementary Fig. 9 | A slight red-shift as the film thickness increases. PL spectra of the 1.4 M, 1.5 M, 1.6 M, 1.8 M and 2.0 M perovskite measured from (a) front and (b) rear side right before corresponding TRPL to confirm the emission position with a structure of glass/perovskite, indicating an emission peak at around 810 nm with a slight red-shift as the strain relax.

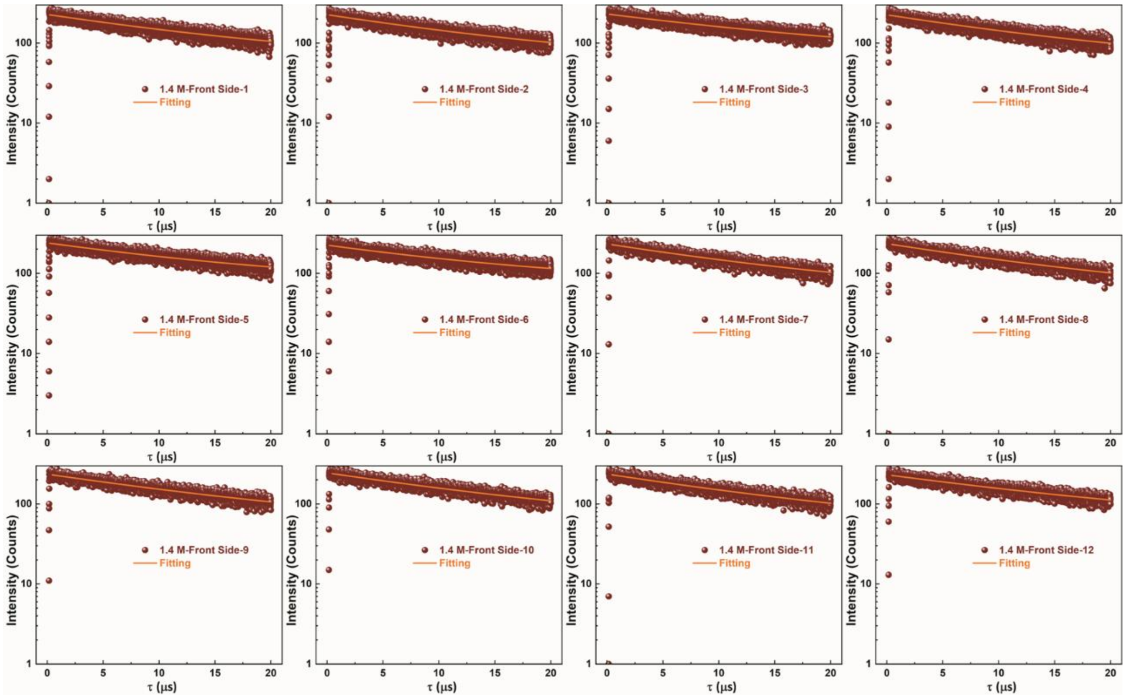

Supplementary Fig. 10 | TRPL spectra of the 1.4 M from front side and it was fitted by single-exponential model. The TRPL was measured by setting the counts peak to be 275.

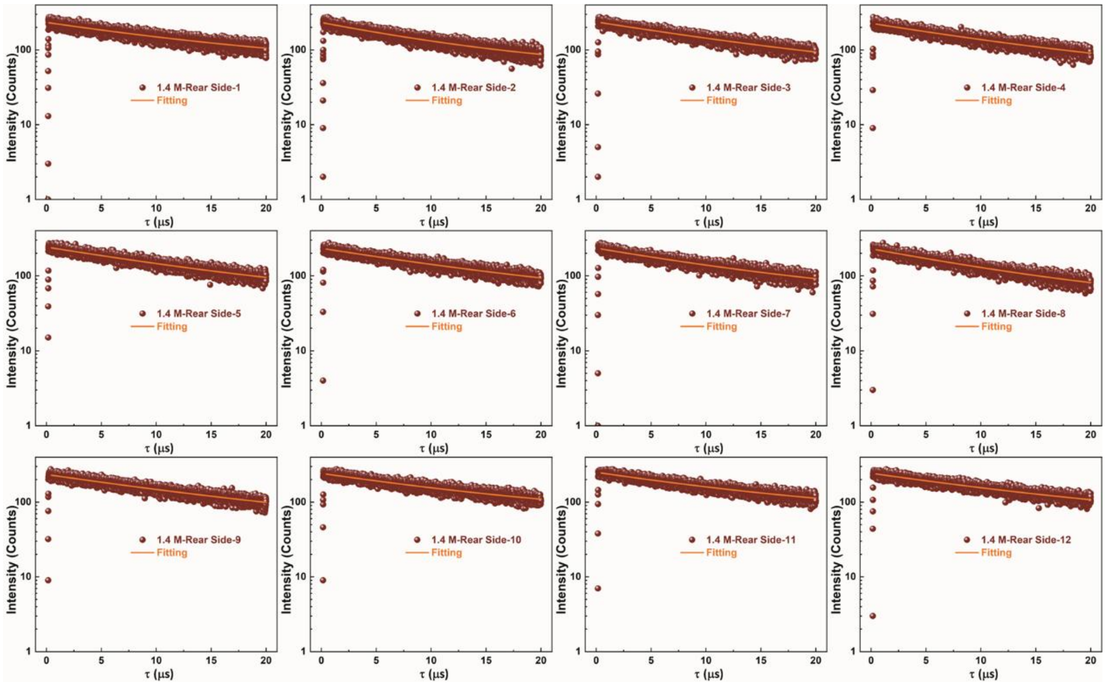

Supplementary Fig. 11 | TRPL spectra of the 1.4 M perovskite from rear side and it was fitted by single-exponential model. The TRPL was measured by setting the counts peak to be 275.

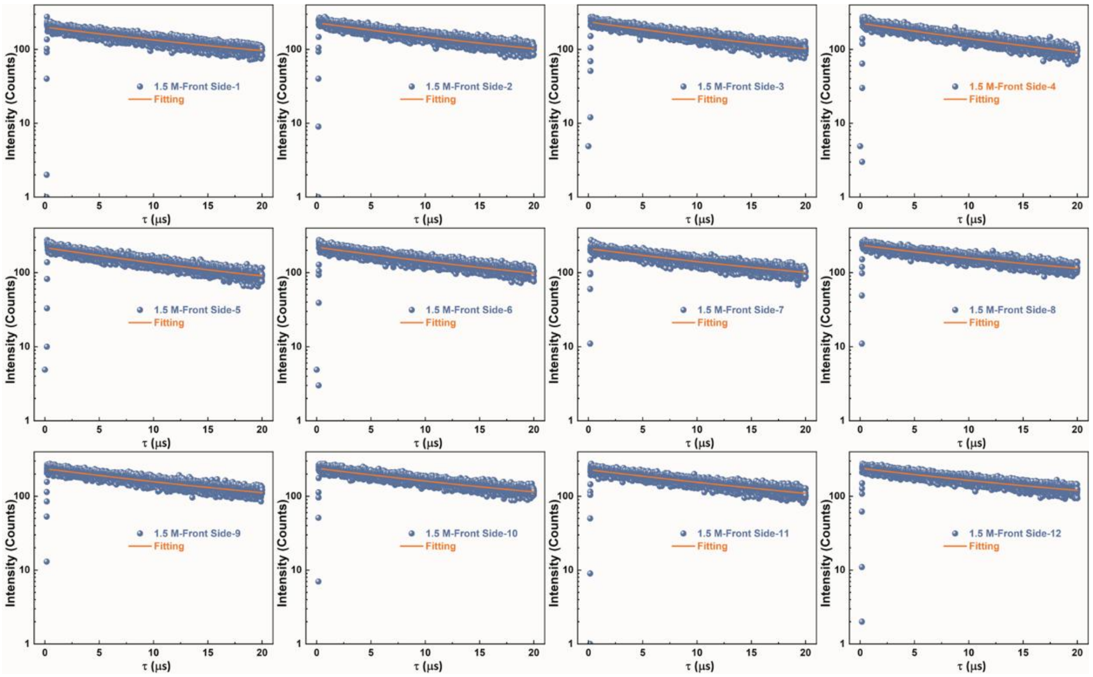

Supplementary Fig. 12 | TRPL spectra of the 1.5 M perovskite from front side and it was fitted by single-exponential model. The TRPL was measured by setting the counts peak to be 275.

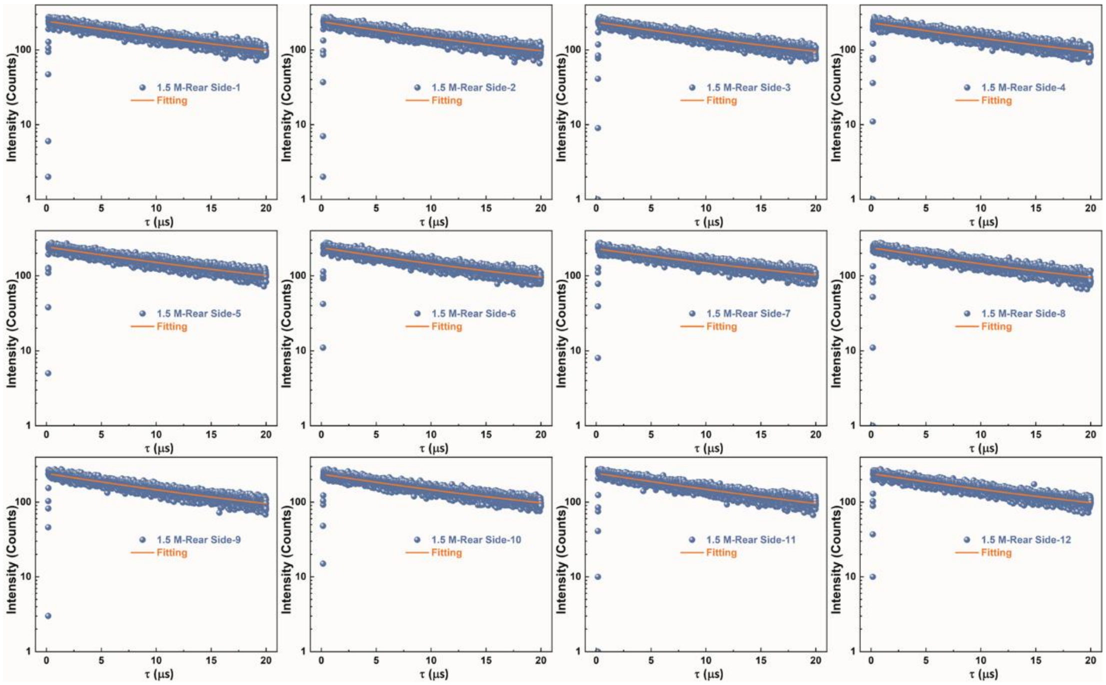

Supplementary Fig. 13 | TRPL spectra of the 1.5 M perovskite from rear side and it was fitted by single-exponential model. The TRPL was measured by setting the counts peak to be 275.

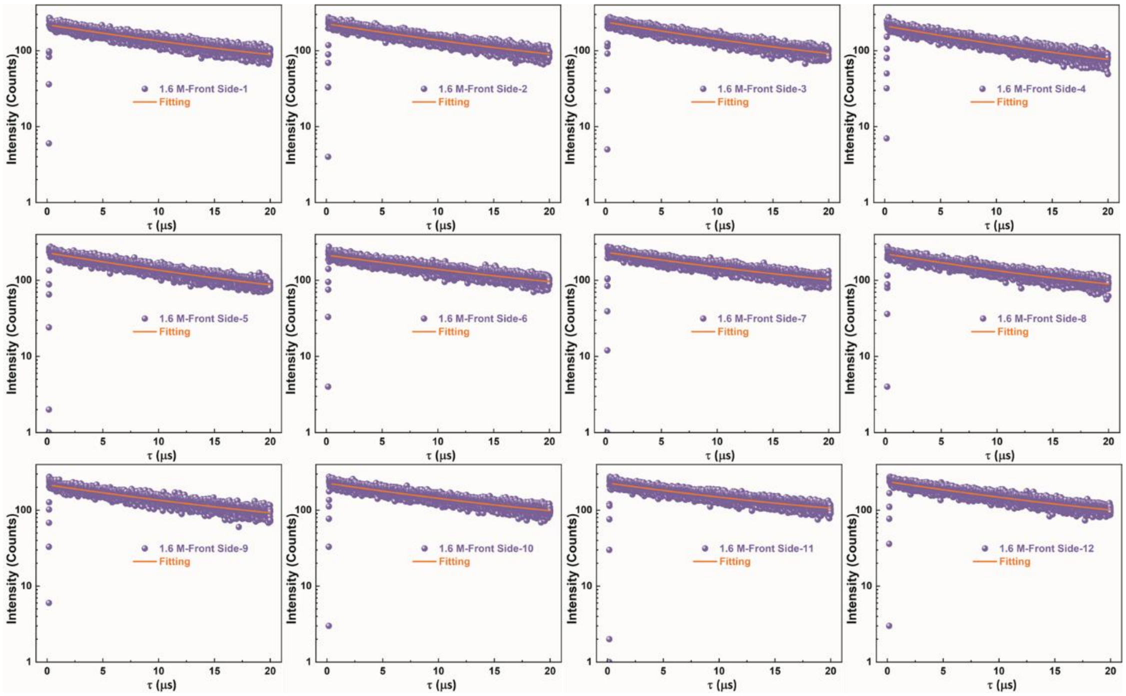

Supplementary Fig. 14 | TRPL spectra of the 1.6 M perovskite from front side and it was fitted by single-exponential model. The TRPL was measured by setting the counts peak to be 275.

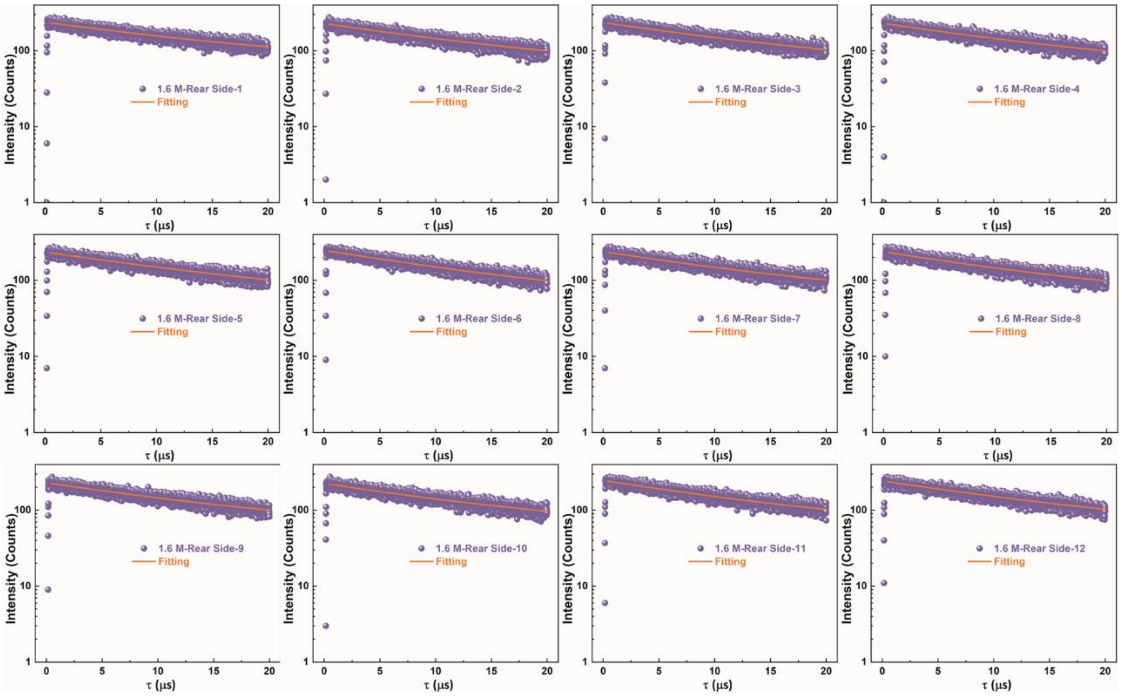

Supplementary Fig. 15 | TRPL spectra of the 1.6 M perovskite from rear side and it was fitted by single-exponential model. The TRPL was measured by setting the counts peak to be 275.

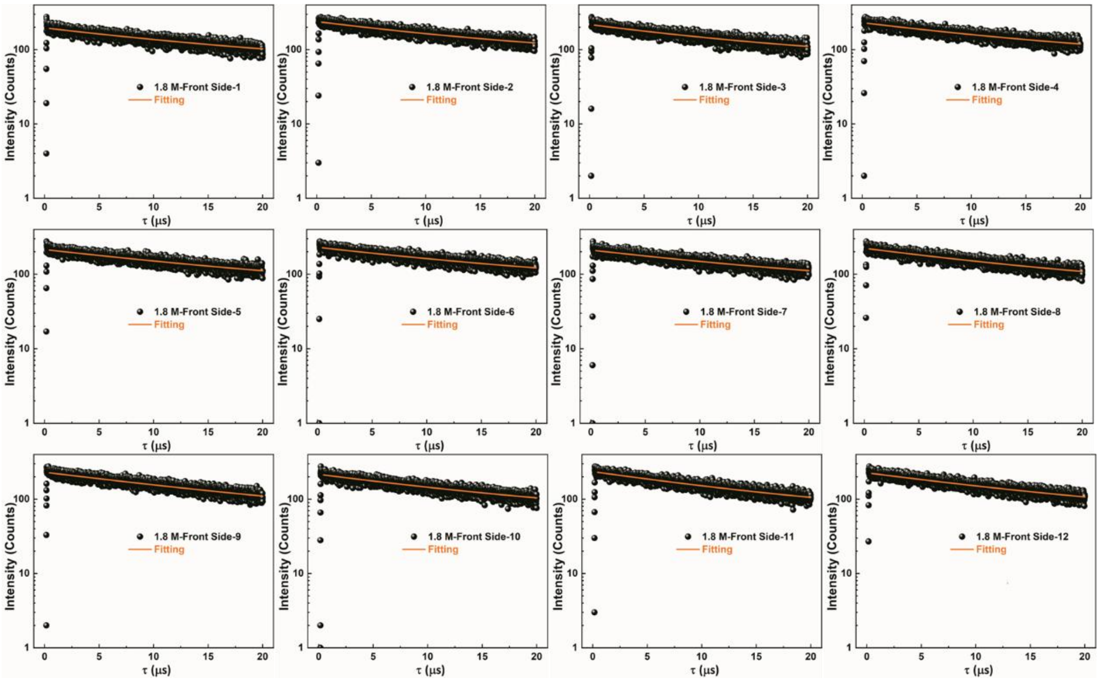

Supplementary Fig. 16 | TRPL spectra of the 1.8 M perovskite from front side and it was fitted by single-exponential model. The TRPL was measured by setting the counts peak to be 275.

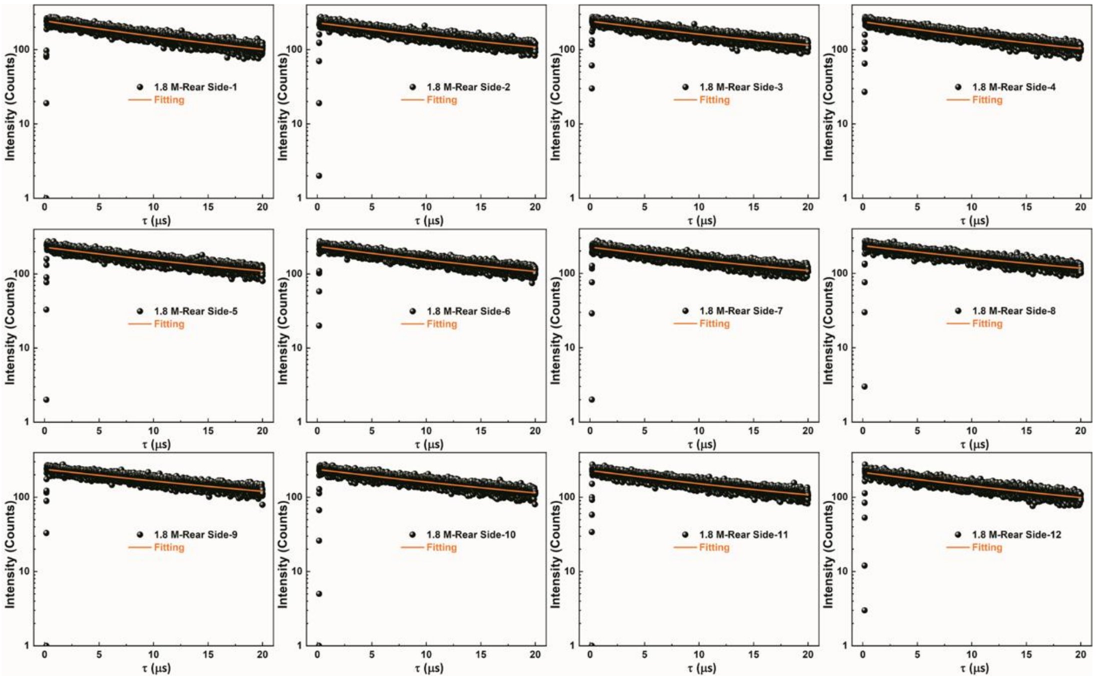

Supplementary Fig. 17 | TRPL spectra of the 1.8 M perovskite from rear side and it was fitted by single-exponential model. The TRPL was measured by setting the counts peak to be 275.

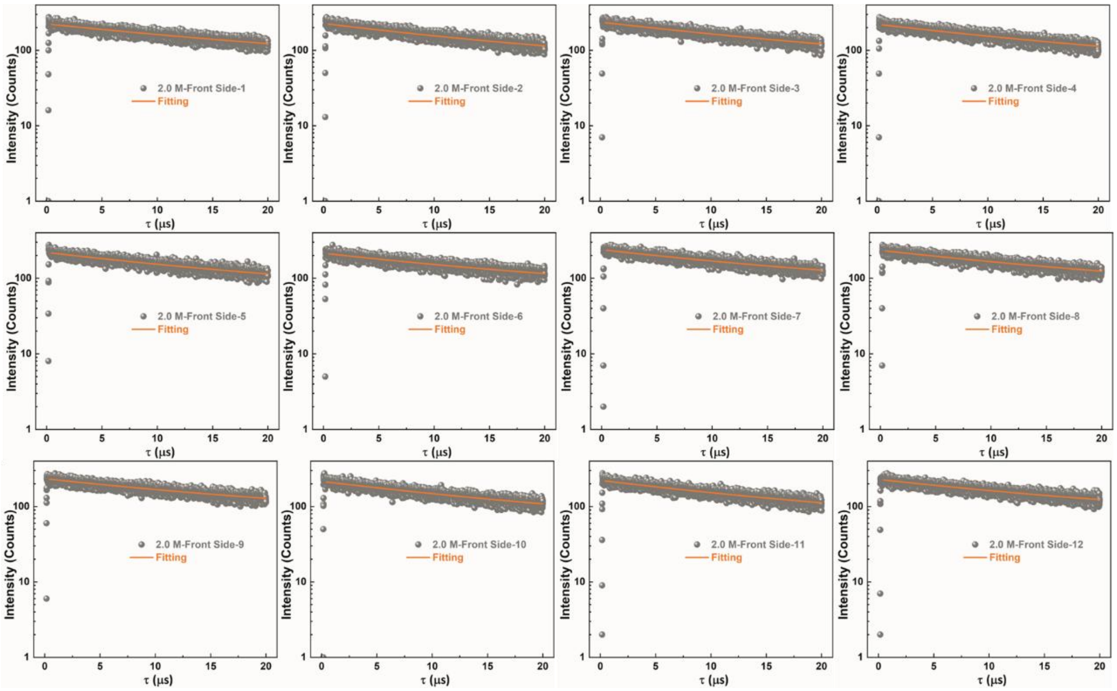

Supplementary Fig. 18 | TRPL spectra of the 2.0 M perovskite from front side and it was fitted by single-exponential model. The TRPL was measured by setting the counts peak to be 275.

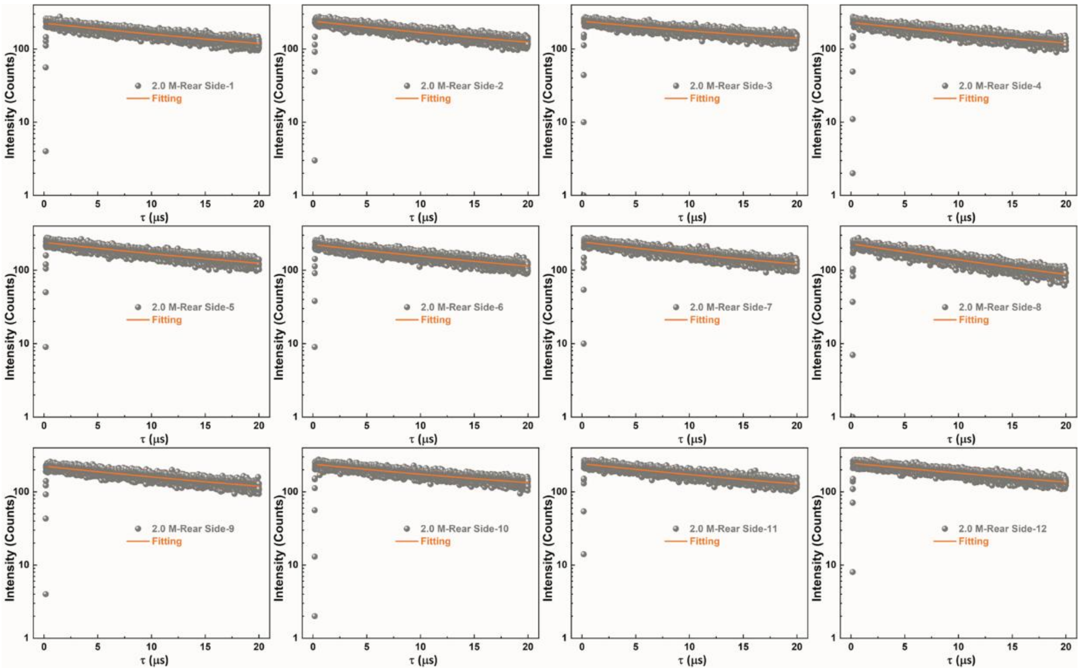

Supplementary Fig. 19 | TRPL spectra of the 2.0 M perovskite from rear side and it was fitted by single-exponential model. The TRPL was measured by setting the counts peak to be 275.

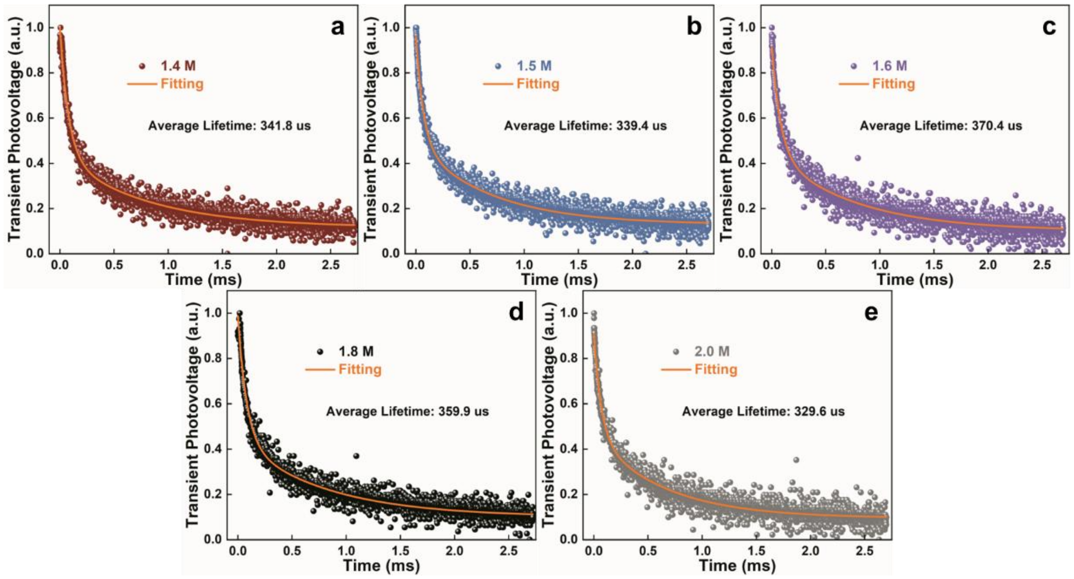

Supplementary Fig. 20 | Negligible variation in the carrier lifetime. Transient photovoltage (TPV) decay of (a) 1.4 M, (b) 1.5 M, (c) 1.6 M, (d) 1.8 M and (e) 2.0 M.

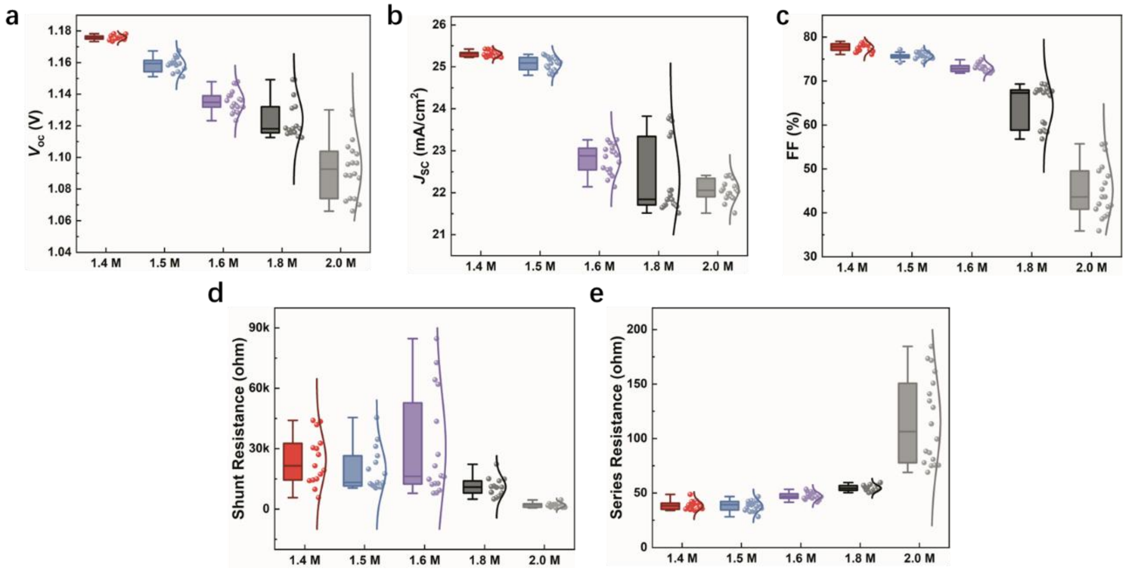

Supplementary Fig. 21 | Successively changed PCE parameters as perovskite film thickness increased. Statistics of the (a) open-circuit voltage, (b) short-circuit current density, (c) Fill Factor, (d) shunt resistance and (e) series resistance for the PSCs with different thickness of perovskite layer. The data was collected from 18 devices for each condition. Centre line, median; box limits, 25th and 75th percentiles; curve, normal distribution curve; whiskers, outliers.

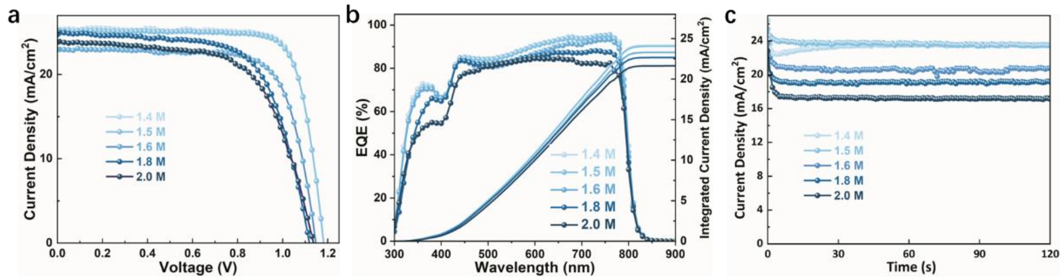

Supplementary Fig. 22 | Successively decreased PCE as perovskite film thickness increased. (a) Current density–voltage curves and (b) EQEs of the PSC devices with different film thickness of perovskite layer. (c) Stabilized maximum power output and the photocurrent density at maximum power point as a function of time for the best performing perovskite solar cells of the 1.4 M, 1.5 M, 1.6 M, 1.8 M and 2.0 M perovskite recorded under simulated one-sun AM1.5G illumination

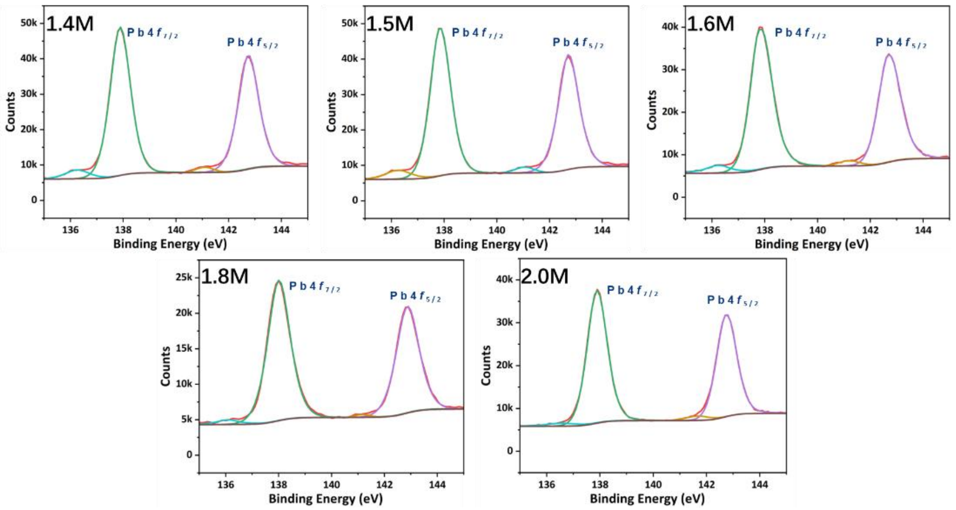

Supplementary Fig. 23 | Examination of perovskite film chemistry. XPS data for Pb 4f 7/2 and Pb 4f 5/2 core-level spectra in the 1.4 M, 1.5 M, 1.6 M, 1.8 M and 2.0 M perovskite films.

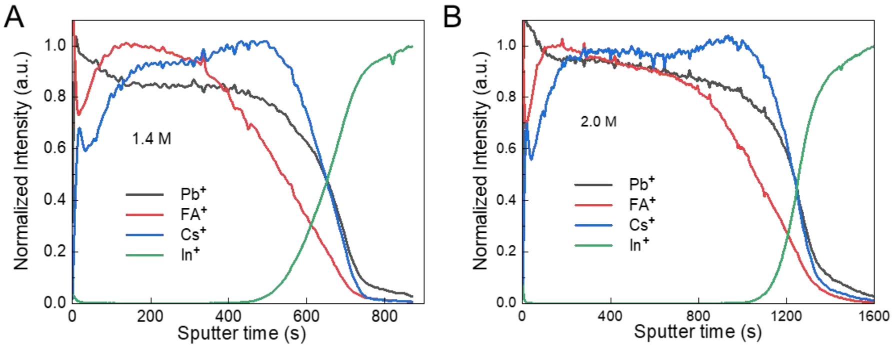

Supplementary Fig. 24 | Comparable compositional distribution profiles. Time of Flight secondary ion mass spectrometry of (A) 1.4 M and (B) 2.0 M perovskite films. ToF-SIMS depth-profile analysis was performed using a PHI NanoTOF III instrument (ULVAC-PHI, Inc.), where a 3 kV Ar ion beam was used for erosion and a 25 keV Bi^+ pulsed primary ion beam was used for the analysis. The area of analysis was 100 × 100 μm^2 while the sputtering area was 400 × 400 μm^2.

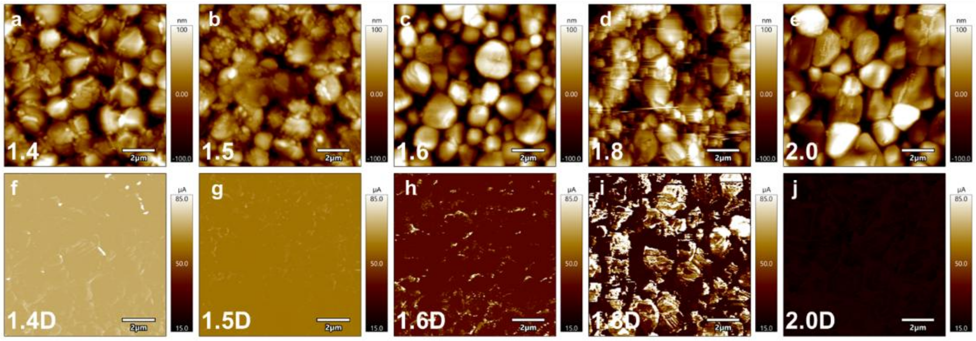

Supplementary Fig. 25 | Investigation on conductivity via conductive AFM. AFM images of 1.4 M (a), 1.5 M (b), 1.6 M (c), 1.8 M (d) and 2.0 M (e) with corresponding CAFM images of 1.4 M (f), 1.5 M (g), 1.6 M (h), 1.8 M (i) and 2.0 M (j).

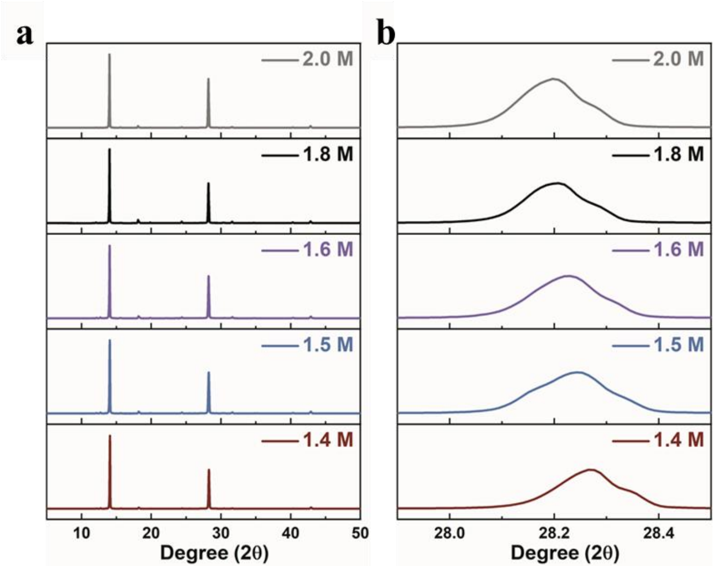

Supplementary Fig. 26 | Comparison of XRD results. (a)XRD patterns of the 1.4 M, 1.5 M, 1.6 M, 1.8 M and 2.0 M perovskites; (b) The enlarged X-ray diffraction patterns of (002) planes.

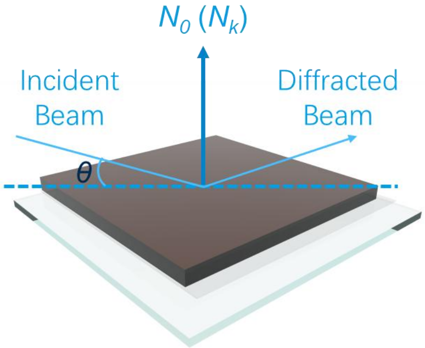

Supplementary Fig. 27 | Schematic illustration of Grazing-Incidence Wide-Angle X-ray Scattering (GIWAXS) measurement. The incident angle (θ) was tuned from 0.3° to 1.0° with a step increase of 0.1° and the X-ray energy used for GIWAXS measurement is 9.9 KeV.

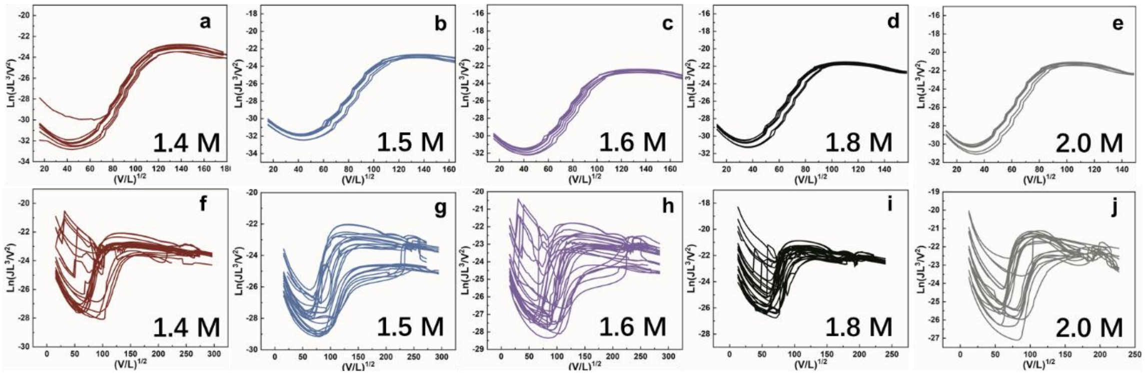

Supplementary Fig. 28 | SCLC curves. SCLC measurements of 1.4 M, 1.5 M, 1.6 M, 1.8 M and 2.0 M for (a-e) electron-only devices (ITO/SnO_2/perovskite/PCBM/Au) and (f-j) hole-only devices (ITO/PEDOT:PSS/perovskite/spiro-OMeTAD/Au). See the detailed measurements and calculation methods in Supplementary Note 3.

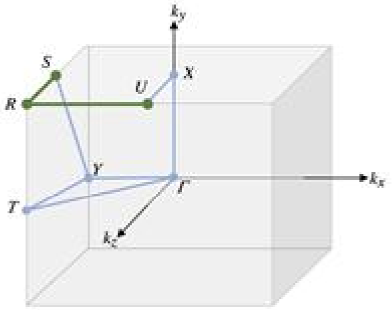

Supplementary Fig. 29 | Numerical differentiation method for DFT calculations. A five-point numerical differentiation method and $S \rightarrow R \rightarrow U$ k-path, including the valence band maximum (VBM) and conduction band minimum (CBM), is used to predict hole and electron m_{eff}.

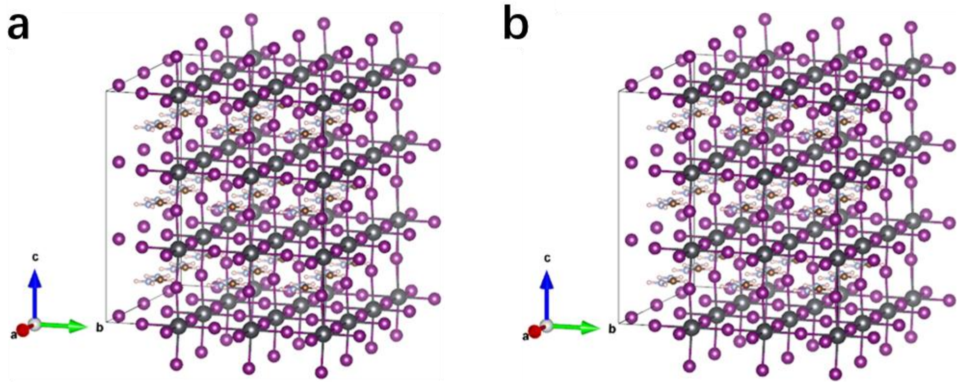

Supplementary Fig. 30 | Supercell structures for DFT calculations: (a) the 1.4 M perovskite and (b) the 2.0 M perovskite. Since the strain we introduced is on the order of 1% (~0.04Angs), the difference in a and b may not be clearly visible.

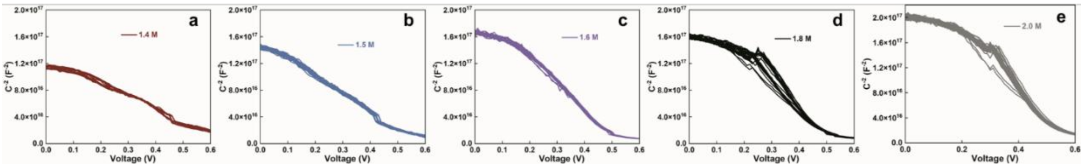

Supplementary Fig. 31 | Capacitance-voltage measurements of 1.4 M (a), 1.5 M (b), 1.6 M (c), 1.8 M (d) and 2.0 M (e) for electron only devices (ITO/SnO_2/perovskite/ PCBM/Au). It was measured by cryogen-free cryogenic probe stations & 4200A-SCS (CRX-4K) with a constant frequency of 1 kHz. See the detailed calculation methods in supplementary Note 5.

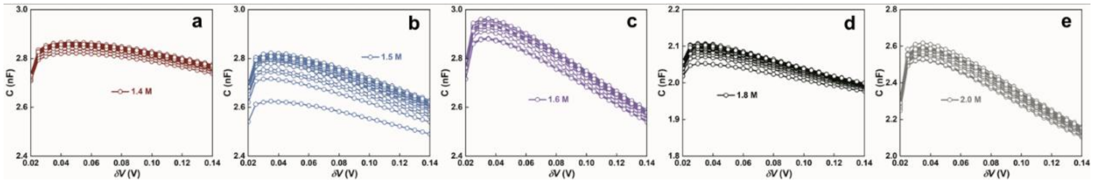

Supplementary Fig. 32 | DLCP spectrum of 1.4 M (a), 1.5 M (b), 1.6 M (c), 1.8 M (d) and 2.0 M (e) for electron only devices (ITO/SnO_2/perovskite/ PCBM/Au) with the frequency of 10K Hz. The detailed method seen the detailed calculation methods in supplementary Note 6.

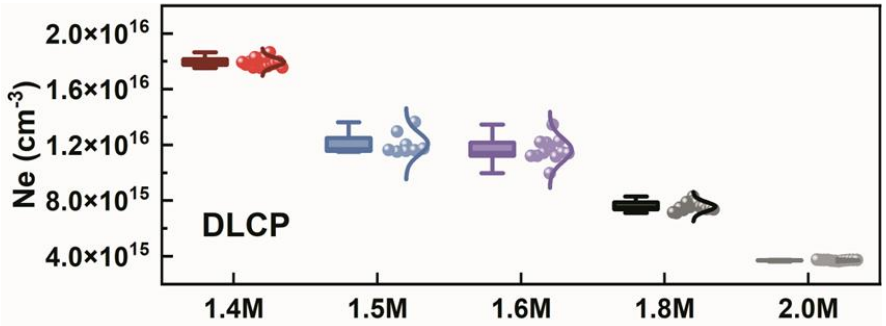

Supplementary Fig. 33 | The extracted electron concentrations from the DLCP measurements for perovskite films with different thicknesses. Centre line, median; box limits, 25th and 75th percentiles; curve, normal distribution curve; whiskers, outliers.

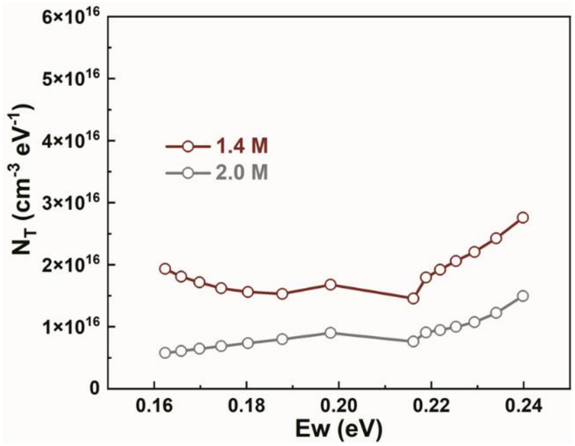

Supplementary Fig. 34 | Trap density of states of 1.4 M and 2.0 M. The detailed calculation method seen Supplementary Note 7.

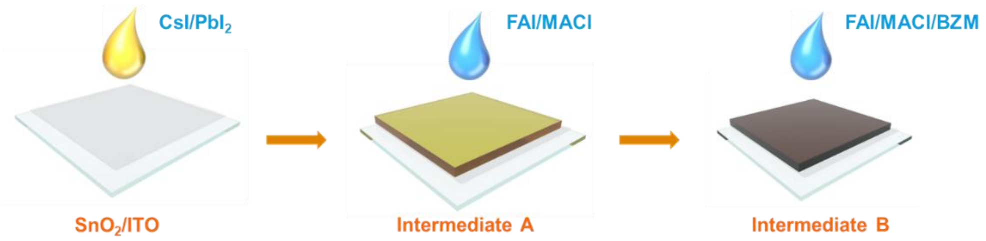

Supplementary Fig. 35 | Schematic illustration of the strain regulation strategy to fabricate thick perovskite films.

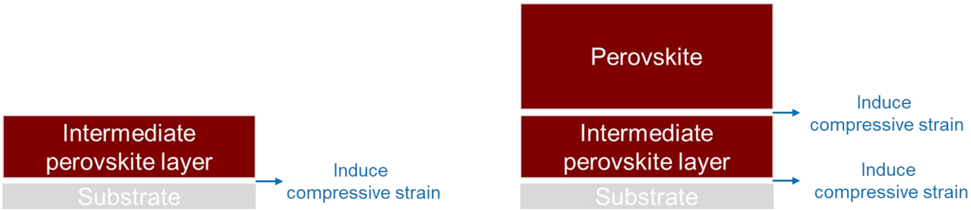

Supplementary Fig. 36 | Schematic illustration of the mechanisms of SRS.

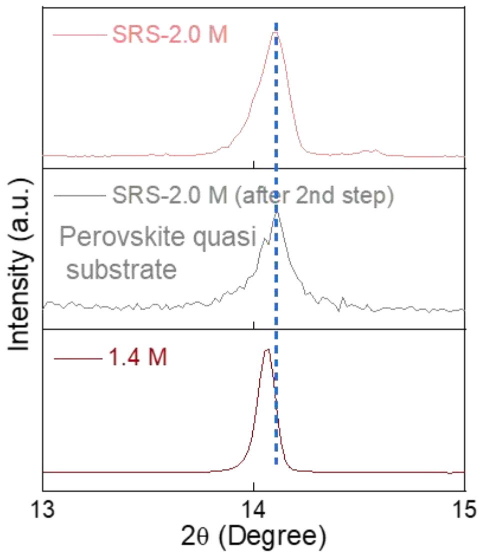

Supplementary Fig. 37 | Strain maintained by the perovskite quasi substrate. X-ray diffraction patterns of the (001) plane for the 1.4 M perovskite thin film, SRS-based 2.0 M perovskite thick film, and the intermediate perovskite layer (the perovskite film fabricated after the first round of dripping of the organic cation precursor in the SRS).

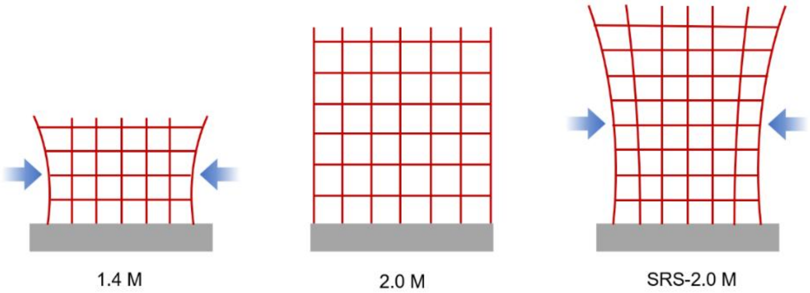

Supplementary Fig. 38 | Schematic illustration of the strain type and distribution in the perovskite films of 1.4 M, 2.0 M and SRS-2.0 M.

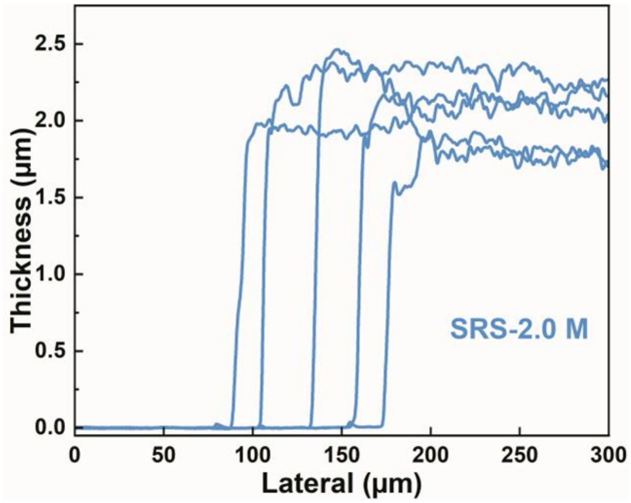

Supplementary Fig. 39 | Film thickness of the perovskite layer deposited via SRS with an average thickness of 2.054 μm.

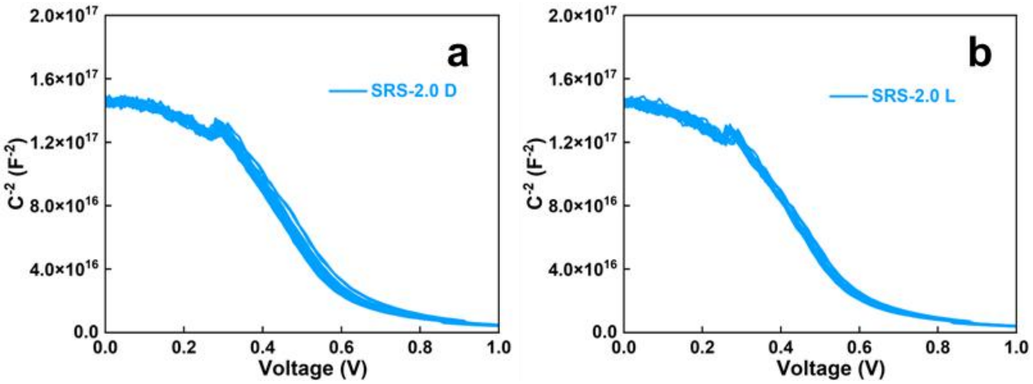

Supplementary Fig. 40 | Capacitance-voltage measurements of SRS-2.0 M. Capacitance-voltage spectrum of SRS-2.0 M under dark condition (a) and light condition (b), abbreviated as SRS-2.0 D and SRS-2.0 L respectively.

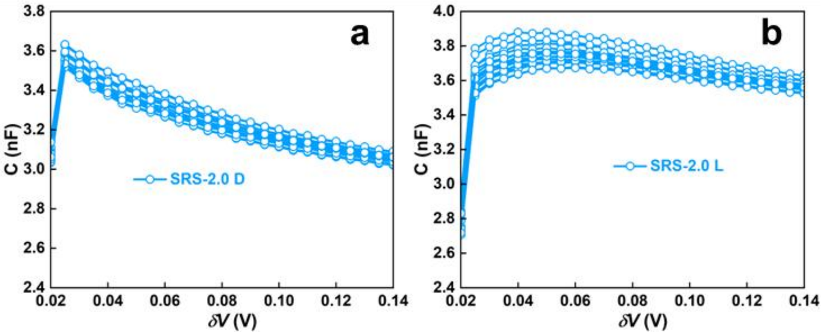

Supplementary Fig. 41 | DLCP measurements of SRS-2.0 M. DLCP spectrum of SRS-2.0 M with the frequency of 10K Hz under dark condition (a) and light condition (b), abbreviated as SRS-2.0 D and SRS-2.0 L respectively.

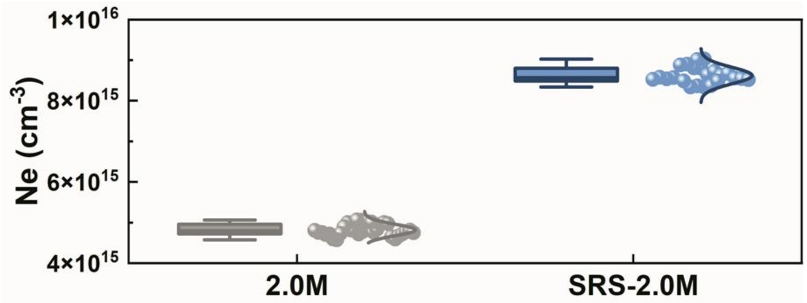

Supplementary Fig. 42 | Statistical results of electron concentration of SRS-2.0 M and 2.0 M extracted from C-V plots under dark condition. Centre line, median; box limits, 25th and 75th percentiles; curve, normal distribution curve; whiskers, outliers.

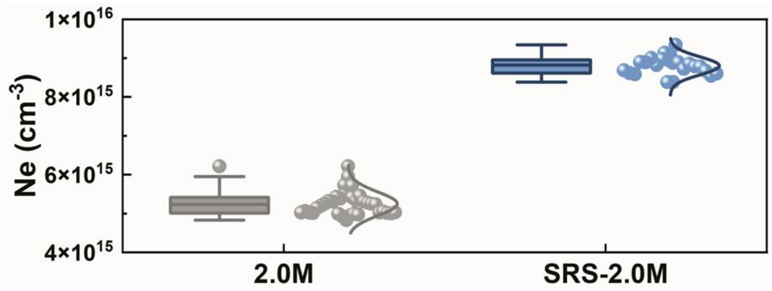

Supplementary Fig. 43 | Statistical results of electron concentration of SRS-2.0 M and 2.0 M extracted from C-V plots under light condition. Centre line, median; box limits, 25th and 75th percentiles; curve, normal distribution curve; whiskers, outliers.

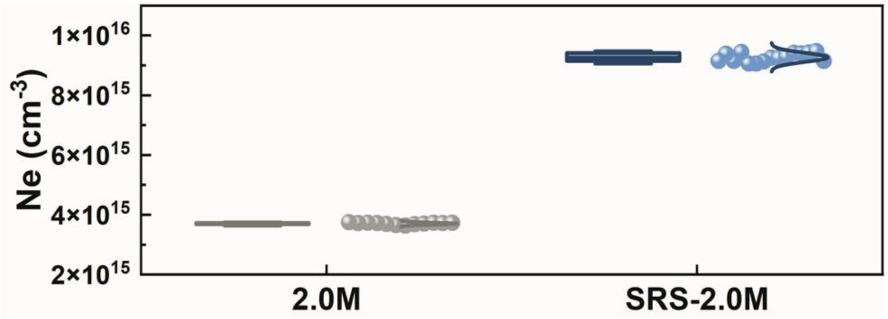

Supplementary Fig. 44 | Statistical results of electron concentration of SRS-2.0 M and 2.0 M extracted from DLCP plots under dark condition. Centre line, median; box limits, 25th and 75th percentiles; curve, normal distribution curve; whiskers, outliers.

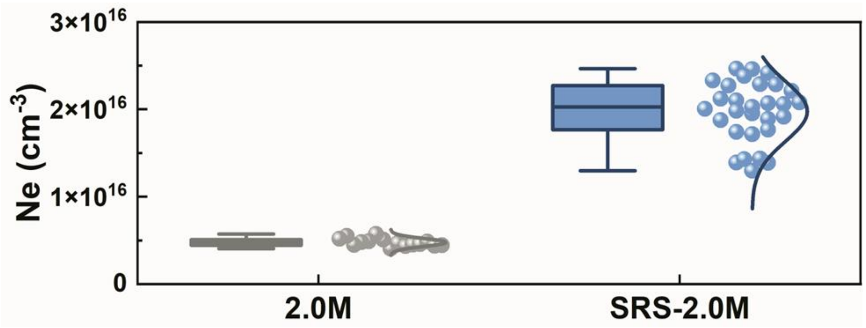

Supplementary Fig. 45 | Statistical results of electron concentration of SRS-2.0 M and 2.0 M extracted from DLCP plots under light condition. Centre line, median; box limits, 25th and 75th percentiles; curve, normal distribution curve; whiskers, outliers.

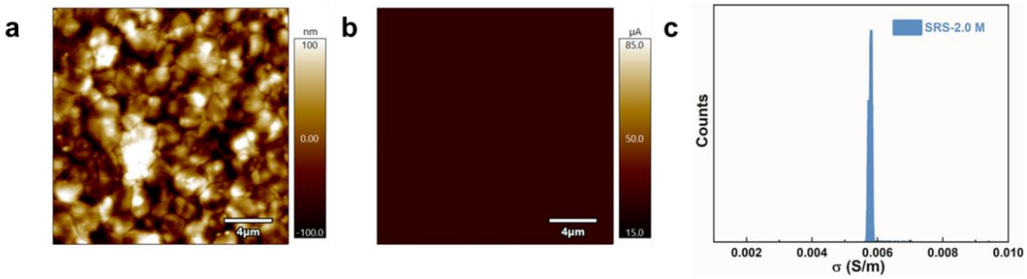

Supplementary Fig. 46 | Investigation on conductivity via conductive AFM. (a) Morphological image of CAFM of SRS-2.0 M with (b) current signals under dark condition. The conductivity extracted from CAFM (c).

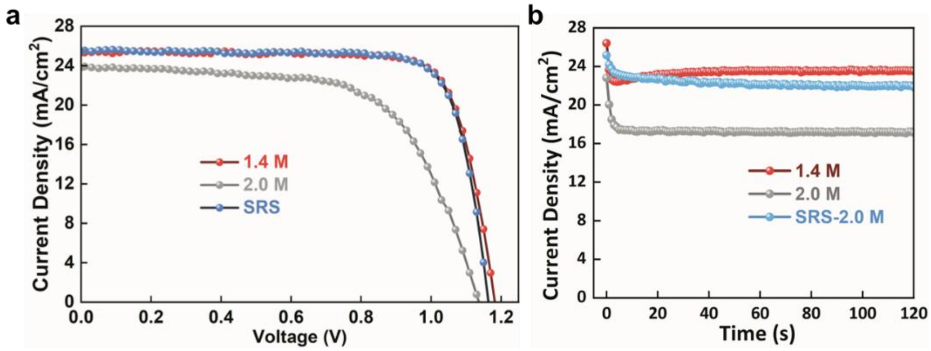

Supplementary Fig. 47 | PCE comparison. (a) *J-V* curves of 1.4 M, 2.0 M and SRS-2.0M perovskites under reverse scan. (b) Stabilized current density of the PSCs biased at V_{max} (0.95 V) with 1.4 M, 2.0 M perovskite and perovskite layer fabricated via SRS-2.0 M.

Report No. 23TR021701

====Measurement Results ====

	Forward Scan (Isc to Voc)	Reverse Scan (Voc to Isc)
Area	0.10 cm^2	
Isc	2.506 mA	2.503 mA
Voc	1.177 V	1.163 V
Pmax	2.251 mW	2.310 mW
Ipm	2.348 mA	2.381 mA
Vpm	0.959 V	0.970 V
FF	76.30 %	79.33 %
Eff	22.51 %	23.10 %

- Active Area was provided by the client.
- Test results listed in this measurement report refer exclusively to the mentioned test sample.
- The results apply only at the time of the test, and do not imply future performance.

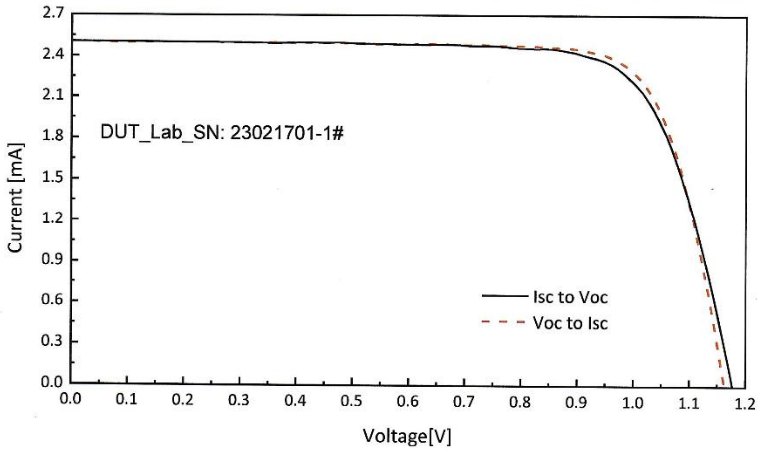

Fig.1 I-V curves of the measured sample

-------End of Report-------

3 / 3

Supplementary Fig. 48 | PCE certification. Certification of the device made with SRS-2.0M by the Shanghai Institute of Microsystem and Information Technology (SIMIT), Chinese Academy of Sciences. Permission to use the logo was obtained from SIMIT.

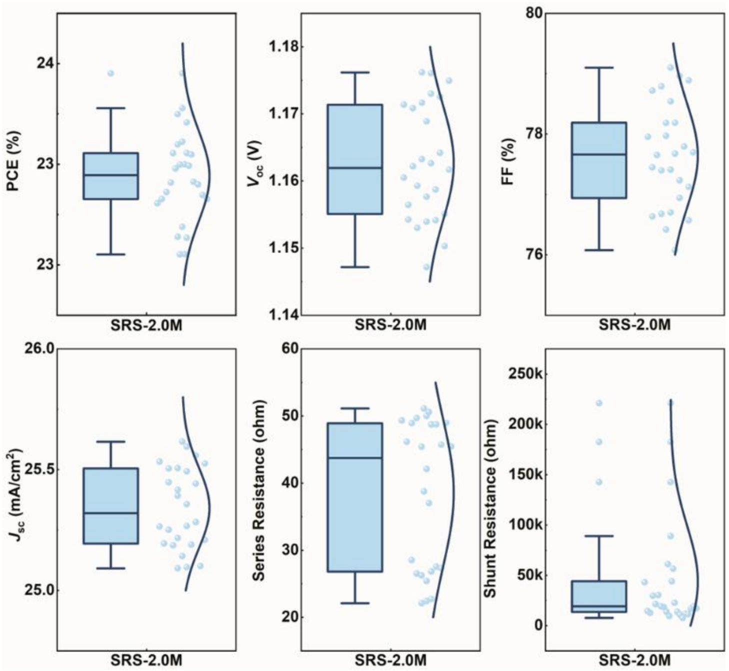

Supplementary Fig. 49 | PCE statistics of SRS-2.0 M. Statistics of PCEs, open-circuit voltage, current density, fill factor, shunt resistance and series resistance of PSCs fabricated via SRS-2.0 M. The data points were collected from 26 devices for each condition. Centre line, median; box limits, 25th and 75th percentiles; curve, normal distribution curve; whiskers, outliers.

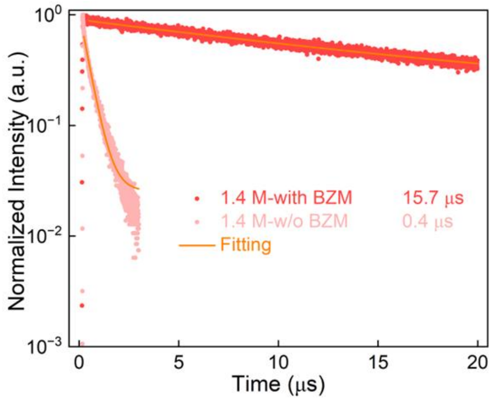

Supplementary Fig. 50 | TRPL plots of 1.4 M perovskite with and without introducing BZM.

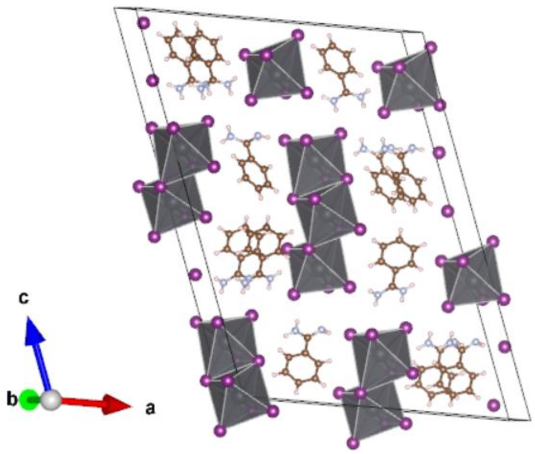

Supplementary Fig. 51 | Crystal structure of the 1D $(BZM)_2Pb_3I_8$ perovskite. The angles between the a and b axes, b and c axes, and a and c axes are 90°, 90°, and 111.253°, respectively.

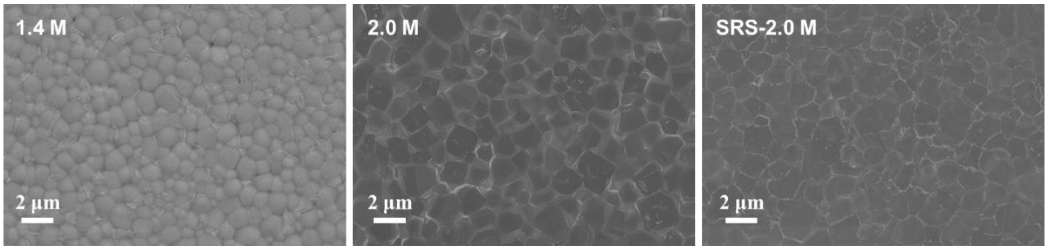

Supplementary Fig. 52 | Comparison of morphologies. Top-view SEM images of 1.4 M, 2.0 M and SRS-2.0 M based perovskite films.

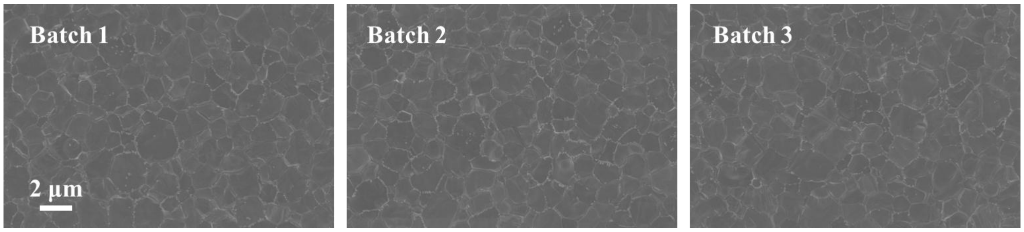

Supplementary Fig. 53 | Morphologies of SRS-2.0 M. Top-view scanning electron microscopy images of the SRS-2.0 M perovskite films from different batches.

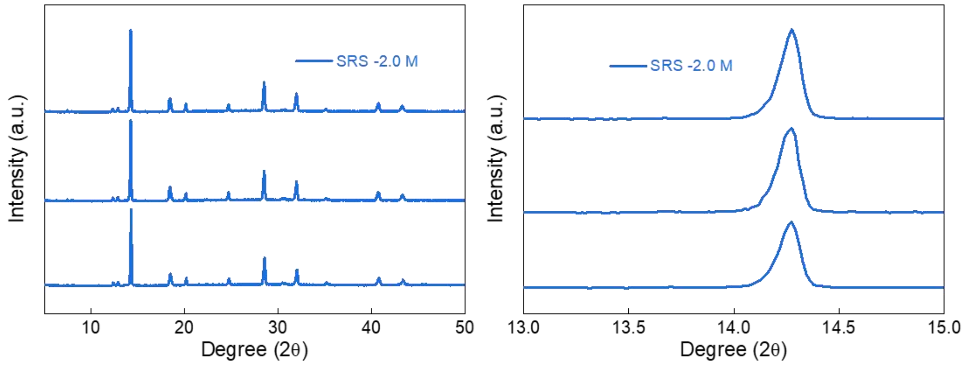

Supplementary Fig. 54 | X-ray diffraction patterns of the SRS-2.0 M perovskite thin films from different batches.

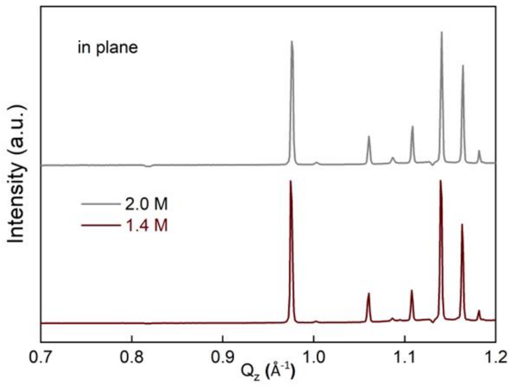

Supplementary Fig. 55 | Integrated GIWAXS patterns with the azimuth angle ranging from 0° to 10° for the 1.4 M and 2.0 M perovskite films. The GIWAXS peaks along the in-plane direction exhibited negligible shift (0.975 $Å^{-1}$ for 1.4 M and 0.976 $Å^{-1}$ for 2.0 M perovskite films).

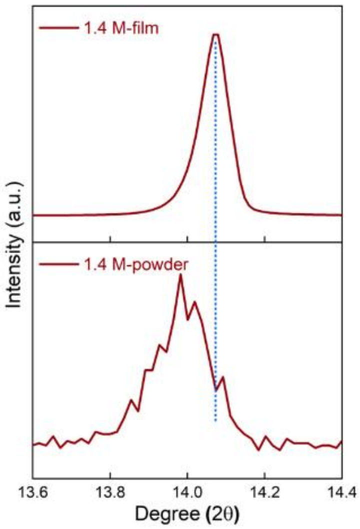

Supplementary Fig. 56 | XRD patterns of the 1.4 M perovskite film and the 1.4 M perovskite powders scraped from the substrate.

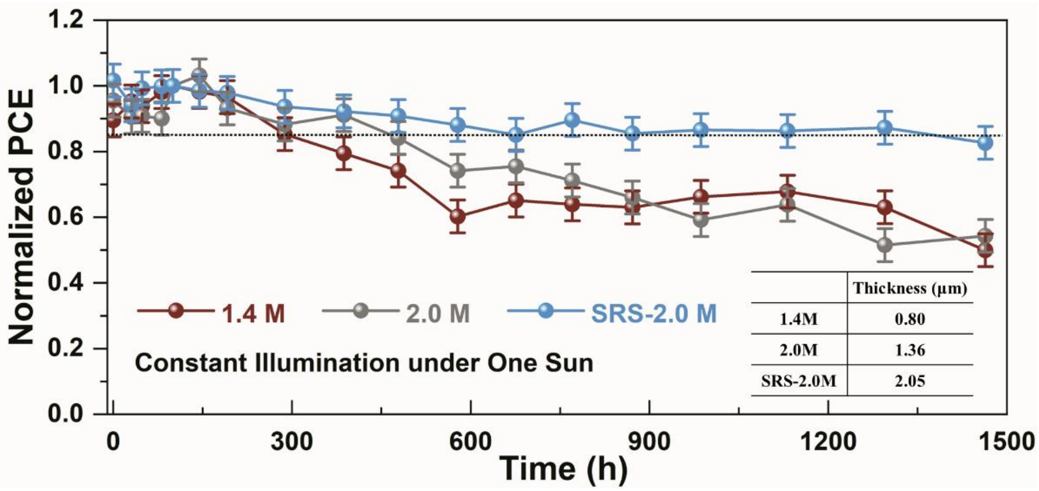

Supplementary Fig. 57 | The PCE tracking of the encapsulated 1.4 M, 2.0 M and SRS-2.0 M perovskite devices under constant illumination ((AM 1.5G)) and 50% relative humidity at open-circuit condition. The corresponding thickness of the perovskite films was summarized in the inserted figure. After 1500 hours continuous illumination at open circuit condition, the devices of SRS 2.0 M still maintain 85% of their original PCEs, whereas the 1.4 M perovskite devices degraded to below 60% of their initial PCEs. Stability statistics were collected from 4 PSCs for each film thicknesses.

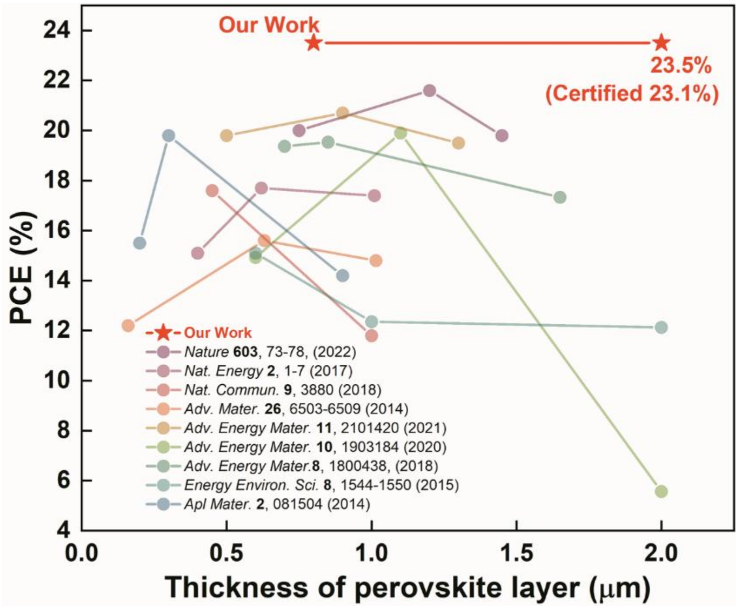

Supplementary Fig. 58 | The published PCE results of thick film perovskite solar cells in comparison with the PCE results in this work.

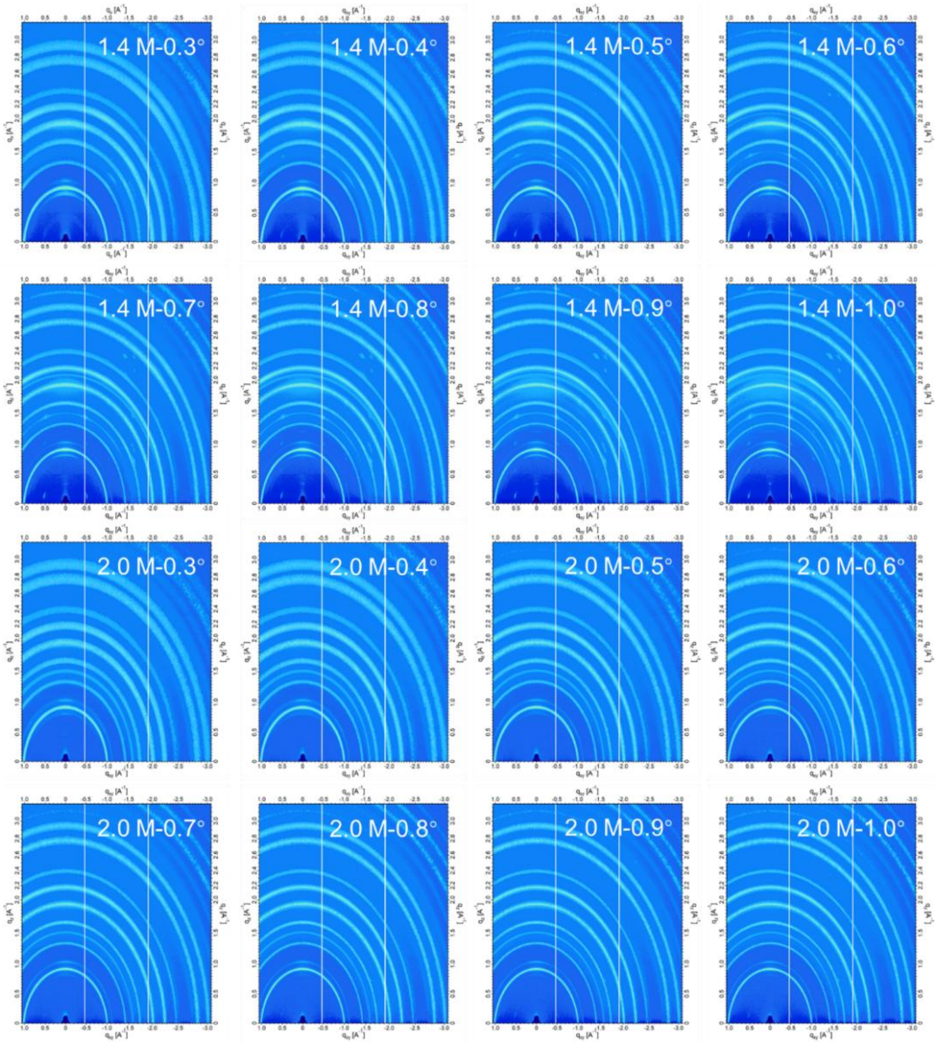

Supplementary Fig. 59 | 2D GIWAXS patterns of perovskite films of 1.4 M and 2.0 M.

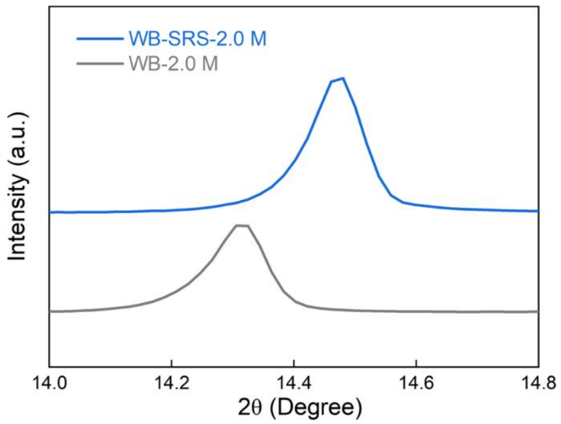

Supplementary Fig. 60 | XRD patterns of 2.0 M and SRS-2.0 M for wide-bandgap perovskite films.

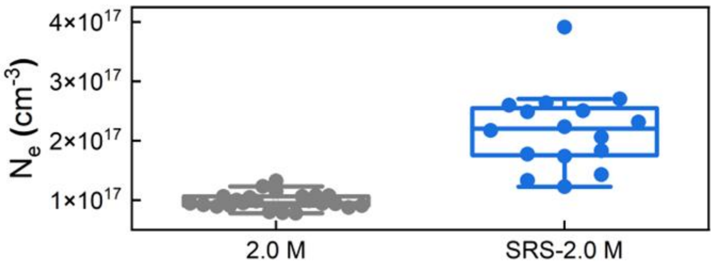

Supplementary Fig. 61 | Statistics of electron concentration of wide bandgap perovskite derived from capacitance-voltage (Mott-Schottky) plot. Centre line, median; box limits, 25th and 75th percentiles; curve, normal distribution curve; whiskers, outliers.

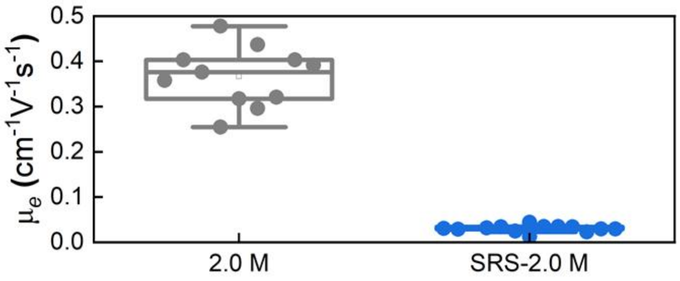

Supplementary Fig. 62 | Statistics of electron mobility of wide bandgap perovskite extracted from space-charge-limited-current (SCLC) measurement. Centre line, median; box limits, 25th and 75th percentiles; curve, normal distribution curve; whiskers, outliers.

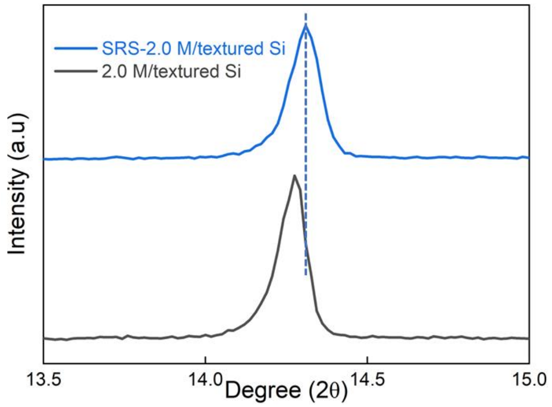

Supplementary Fig. 63 | (001) plane of XRD patterns of SRS-2.0 M and 2.0 M that deposited on the textured Si.

Supplementary Table 1 | Summary of the carrier lifetime extracted from TRPL spectra measured form the rear side of the films

Rear Side (µs)					
	**1.4 M**	**1.5 M**	**1.6 M**	**1.8 M**	**2.0 M**
1	15.5	16.5	16.9	16.2	20.0
2	12.2	15.6	16.1	18.5	20.7
3	16.1	15.7	16.0	17.8	20.0
4	15.5	17.3	16.0	16.3	21.5
5	15.6	16.3	17.0	18.2	19.8
6	16.0	15.4	15.3	18.6	20.3
7	14.5	17.1	15.0	17.8	19.6
8	13.6	15.5	15.1	19.5	17.2
9	17.3	15.8	15.9	21.3	21.6
10	15.8	15.9	15.3	19.3	21.6
11	15.4	16.9	15.0	18.2	21.6
12	16.8	16.5	16.5	18.6	24.1

Supplementary Table 2 | Summary of the carrier lifetime extracted from TRPL spectra measured form the front side of the films

Front Side (μs)					
	**1.4 M**	**1.5 M**	**1.6 M**	**1.8 M**	**2.0 M**
1	16.6	18.4	15.6	18.5	22.0
2	16.3	17.1	15.7	16.8	18.1
3	18.3	16.1	14.8	16.3	20.5
4	15.8	17.5	14.0	17.6	19.8
5	16.4	16.5	15.0	20.4	19.1
6	15.9	18.2	18.3	19.1	19.2
7	16.0	17.9	16.3	18.8	18.9
8	16.3	17.4	14.3	17.9	19.9
9	17.5	18.3	16.2	20.2	19.2
10	17.0	18.6	16.0	17.9	19.1
11	16.9	20.1	16.1	16.6	18.8
12	18.8	18.7	15.9	18.4	19.4

Supplementary Table 3 | EQEs and steady-state outputs of PSCs with different perovskite thickness

		1.4 M	1.5 M	1.6 M	1.8 M	2.0 M
J_{EQE} (mA/cm^2)		24.16	24.04	23.30	22.66	21.63
Best PCE (%)		23.5	23.3	19.1	18.4	17.0
Stead Output	Voltage (V)	0.95	0.97	0.93	0.91	0.91
	Current Density (mA/cm^2)	23.43	23.67	20.65	19.10	17.31
	Stabilized PCE (%)	22.3	23.0	19.2	17.3	15.8

Supplementary Table 4 | Device performance of PSCs with different perovskite thickness

	**1.4 M**	**1.5 M**	**1.6 M**	**1.8 M**	**2.0 M**
**Open Circuit Voltage (V)**	1.18	1.18	1.15	1.12	1.14
**Short Circuit Current Density (mA/cm^2)**	25.38	25.18	22.98	24.79	23.84
**Fill Factor (%)**	78.51	78.46	73.20	66.67	63.41
**Best Power Conversion Efficiency (%)**	23.5	23.3	19.1	18.4	17.0
**Average PCE (%)**	23.1	21.9	18.7	15.8	10.9
**Average FF**	0.77	0.75	0.73	0.64	0.45
**Average Rs (ohm)**	38.5	38.3	47.2	65.6	116.0

Supplementary Table 5 | Electron mobility measured by SCLC method

**Electron mobility μ_e(cm^2 V^{-1} s^{-1})**				
**1.4 M**	**1.5 M**	**1.6 M**	**1.8 M**	**2.0 M**
10.5	9.6	28.5	79.2	137.2
8.8	32.4	34.2	64.3	98.7
3.4	17.7	14.1	61.4	78.4
2.5	12.2	11.4	54.4	70.2
10.5	20.3	51.9	49.8	66.8
10.5	24.5	27.6	32.7	24.1
7.4	25.7	35.6	9.0	17.8
5.2	18.5	26.5	6.0	10.6

Supplementary Table 6 | Hole mobility measured by SCLC method

**Hole mobility μ_h ($cm^2 V^{-1} s^{-1}$)**				
**1.4 M**	**1.5 M**	**1.6 M**	**1.8 M**	**2.0 M**
0.830	1.266	0.929	1.506	1.407
0.700	1.100	0.892	1.456	1.100
0.700	0.800	0.758	1.456	1.024
0.685	0.723	0.725	1.407	0.800
0.682	0.696	0.697	1.361	0.774
0.609	0.696	0.444	1.361	0.774
0.593	0.605	0.427	1.310	0.673
0.575	0.585	0.427	1.183	0.632
0.545	0.489	0.392	1.152	0.569
0.501	0.469	0.390	1.150	0.491
0.487	0.230	0.384	0.860	0.431
0.447	0.213	0.368	0.805	0.370
0.419	0.178	0.313	0.672	0.334
0.329	0.137	0.299	0.565	0.268
0.270	0.121	0.294	0.186	0.184
0.221	0.094	0.283	0.184	0.152
0.213	0.069	0.212	0.040	0.144
0.113	0.062	0.051	0.036	0.116
0.008	0.058	0.038	0.034	0.091
0.005	0.054	0.013	0.022	0.052
0.004	0.015	0.011	0.016	0.035
0.001	0.010	0.006	0.015	0.028

Supplementary Table 7 | Thickness of perovskite films

	**1.4 M**	**1.5 M**	**1.6 M**	**1.8 M**	**2.0 M**
**Thickness (μm)**	0.63	0.71	0.79	1.46	1.43
	0.86	0.90	0.96	1.03	1.33
	0.99	0.87	0.91	1.34	1.36
	0.80	0.92	0.86	1.29	1.17
	0.75	0.65	0.82	0.96	1.51
**Average (μm)**	0.80	0.81	0.87	1.22	1.36

Supplementary Table 8 | Carrier effective mass calculated by DFT

Effective Mass (m_0)		
	**Hole**	**Electron**
**1.4 M**	0.215	0.103
**2.0 M**	0.228	0.075

Supplementary Table 9 | Electron concentration (cm^{-3}) derived from CV plots

1.4 M	1.5 M	1.6 M	1.8 M	2.0 M
$1.79*10^{16}$	$1.20*10^{16}$	$7.54*10^{15}$	$8.26*10^{15}$	$5.06*10^{15}$
$1.76*10^{16}$	$1.19*10^{16}$	$7.49*10^{15}$	$7.88*10^{15}$	$5.05*10^{15}$
$1.75*10^{16}$	$1.18*10^{16}$	$7.45*10^{15}$	$7.11*10^{15}$	$5.02*10^{15}$
$1.75*10^{16}$	$1.18*10^{16}$	$7.39*10^{15}$	$6.91*10^{15}$	5.02E+15
$1.74*10^{16}$	$1.16*10^{16}$	$7.38*10^{15}$	$6.82*10^{15}$	$5.01*10^{15}$
$1.73*10^{16}$	$1.15*10^{16}$	$7.37*10^{15}$	$6.76*10^{15}$	$5.00*10^{15}$
$1.72*10^{16}$	$1.15*10^{16}$	$7.36*10^{15}$	$6.62*10^{15}$	$5.00*10^{15}$
$1.72*10^{16}$	$1.15*10^{16}$	$7.35*10^{15}$	$6.59*10^{15}$	$4.97*10^{15}$
$1.71*10^{16}$	$1.15*10^{16}$	$7.34*10^{15}$	$6.47*10^{15}$	$4.96*10^{15}$
$1.71*10^{16}$	$1.14*10^{16}$	$7.33*10^{15}$	$6.45*10^{15}$	$4.89*10^{15}$
$1.71*10^{16}$	$1.14*10^{16}$	$7.32*10^{15}$	$6.43*10^{15}$	$4.86*10^{15}$
$1.71*10^{16}$	$1.14*10^{16}$	$7.32*10^{15}$	$6.42*10^{15}$	$4.85*10^{15}$
$1.70*10^{16}$	$1.14*10^{16}$	$7.30*10^{15}$	$6.39*10^{15}$	$4.83*10^{15}$
$1.69*10^{16}$	$1.13*10^{16}$	$7.30*10^{15}$	$6.39*10^{15}$	$4.81*10^{15}$
$1.69*10^{16}$	$1.13*10^{16}$	$7.28*10^{15}$	$6.38*10^{15}$	$4.81*10^{15}$
$1.68*10^{16}$	$1.12*10^{16}$	$7.27*10^{15}$	$6.28*10^{15}$	$4.81*10^{15}$
$1.57*10^{16}$	$1.12*10^{16}$	$7.24*10^{15}$	$6.09*10^{15}$	$4.80*10^{15}$
$1.49*10^{16}$	$1.11*10^{16}$	$7.23*10^{15}$	$5.64*10^{15}$	$4.77*10^{15}$
$1.45*10^{16}$	$1.11*10^{16}$	$7.19*10^{15}$	$5.56*10^{15}$	$4.77*10^{15}$
$1.44*10^{16}$	$1.11*10^{16}$	$7.18*10^{15}$	$5.48*10^{15}$	$4.76*10^{15}$
$1.43*10^{16}$	$1.11*10^{16}$	$7.16*10^{15}$	$5.45*10^{15}$	$4.76*10^{15}$
$1.42*10^{16}$	$1.10*10^{16}$	$7.13*10^{15}$	$5.37*10^{15}$	$4.76*10^{15}$

Supplementary References

1. R. Wang *et al.*. Unraveling the surface state of photovoltaic perovskite thin film. *Matter* **4**, 2417-2428 (2021).
2. C. Zhu *et al.*. Strain engineering in perovskite solar cells and its impacts on carrier dynamics. *Nat. Commun.* **10**, 815 (2019).
3. J. Xue *et al.*. Crystalline Liquid-like Behavior: Surface-Induced Secondary Grain Growth of Photovoltaic Perovskite Thin Film. *J. Am. Chem. Soc.* **141**, 13948-13953 (2019).
4. J. P. Perdew *et al.*. Restoring the Density-Gradient Expansion for Exchange in Solids and Surfaces. *Phys. Rev. Lett.* **100**, 136406 (2008).
5. K. B., J.P. Perdew, M. Ernzerhof. Generalized Gradient Approximation Made Simple. *Phys. Rev. Lett.* **77**, 3865 (1996).
6. S. Grimme, J. Antony, S. Ehrlich, H. Krieg. A consistent and accurate ab initio parametrization of density functional dispersion correction (DFT-D) for the 94 elements H-Pu. *J. Chem. Phys.* **132**, 154104 (2010).
7. S. Grimme. Semiempirical GGA-Type Density Functional Constructed with a Long-Range Dispersion Correction. *J. Comput. Chem.* **27**, 1787-1799 (2006).
8. J. F., G. Kresse. Efficient iterative schemes for ab initio total-energy calculations using a plane-wave basis set. *Phys. Rev. B* **54**, 11169 (1996).
9. C. Freysoldt, J. Neugebauer, C.G. Van de Walle. Fully Ab Initio Finite-Size Corrections for Charged-Defect Supercell Calculations. *Phys. Rev. Lett.* **102**, 016402 (2009).
10. N. Li *et al.*. Cation and anion immobilization through chemical bonding enhancement with fluorides for stable halide perovskite solar cells. *Nat. Energy* **4**, 408-415 (2019).
11. V. M. Le Corre *et al.*. Revealing Charge Carrier Mobility and Defect Densities in Metal Halide Perovskites via Space-Charge-Limited Current Measurements. *ACS Energy Lett.* **6**, 1087-1094 (2021).
12. Zhenyi Ni, C. B., Ye Liu, Qi Jiang, Wu-Qiang Wu, Shangshang Chen, Xuezeng Dai, Bo Chen, Barry Hartweg, Zhengshan Yu, Zachary Holman, Jinsong Huang. Resolving spatial and energetic distributions of trapstates in metal halide perovskite solar cells. *Science* **367,** 1352-1358(2020).
13. Li, N. et al. Cation and anion immobilization through chemical bonding enhancement with fluorides for stable halide perovskite solar cells. *Nat. Energy* **4**, 408-415 (2019).
