# Supplementary material for: Strain regulates the photovoltaic performance of thick-film perovskites
Source: Nat Commun. 2024 Mar 22;15:2579. doi: 10.1038/s41467-024-47019-8 (PMC10960009; doi:10.1038/s41467-024-47019-8)
Supplement: Supplementary file 3 — Reporting Summary [file 41467_2024_47019_MOESM3_ESM.pdf]

nature portfolio

Corresponding author(s): Jingjing Xue, Rui Wang and Yang Yang

Solar Cells Reporting Summary

Nature Portfolio wishes to improve the reproducibility of the work that we publish. This form is intended for publication with all accepted papers reporting the characterization of photovoltaic devices and provides structure for consistency and transparency in reporting. Some list items might not apply to an individual manuscript, but all fields must be completed for clarity.

For further information on Nature Research policies, including our data availability policy, see Authors & Referees.

Experimental design

Please check the following details are reported in the manuscript, and provide a brief description or explanation where applicable.

1. Dimensions

Area of the tested solar cells

- [x] Yes
- [ ] No

0.1 cm2

Explain why this information is not reported/not relevant.

Method used to determine the device area

- [x] Yes
- [ ] No

the aperture area of the device is 0.1cm2, designated by the shadow mask

Explain why this information is not reported/not relevant.

2. Current-voltage characterization

Current density-voltage (J-V) plots in both forward and backward direction

- [x] Yes
- [ ] No

Figure 4e

Voltage scan conditions

- [x] Yes
- [ ] No

reverse, 1.25 V-0 V, step 0.02 V
forward, 0 V-1.25 V, step 0.02 V

Explain why this information is not reported/not relevant.

Test environment

- [x] Yes
- [ ] No

25 ?, N2 gloveboxes

Explain why this information is not reported/not relevant.

Protocol for preconditioning of the device before its characterization

- [ ] Yes
- [x] No

Provide a description of the protocol.

All the devices were measured without pre-conditioning such as light-soaking and applied a bias voltag

Stability of the J-V characteristic

- [x] Yes
- [ ] No

Supplementary Fig. 22C

Explain why this information is not reported/not relevant.

3. Hysteresis or any other unusual behaviour

Description of the unusual behaviour observed during the characterization

- [x] Yes
- [ ] No

the hysteresis is 0.6% as presented in the certification in Supplementary Fig. 46

Explain why this information is not reported/not relevant.

Related experimental data

- [x] Yes
- [ ] No

Supplementary Fig. 46

Explain why this information is not reported/not relevant.

4. Efficiency

External quantum efficiency (EQE) or incident photons to current efficiency (IPCE)

- [x] Yes
- [ ] No

Steady-state power conversion efficiency was calculated by measuring stabilized photocurrent density under a constant bias voltage. External quantum efficiencies (EQEs) were measured using an integrated system (Enlitech) and a lock-in amplifier with a current preamplifier under short-circuits' condition.

Explain why this information is not reported/not relevant.

A comparison between the integrated response under the standard reference spectrum and the response measure under the simulator

☒ Yes ☐ No

Supplementary Fig. 22

Explain why this information is not reported/not relevant.

For tandem solar cells, the bias illumination and bias voltage used for each subcell

☐ Yes ☒ No

Provide a description of the measurement conditions.

This paper focused on single junction perovskite solar cells

5. Calibration

Light source and reference cell or sensor used for the characterization

☒ Yes ☐ No

Si solar cell

Explain why this information is not reported/not relevant.

Confirmation that the reference cell was calibrated and certified

☒ Yes ☐ No

Enlitech

Explain why this information is not reported/not relevant.

Calculation of spectral mismatch between the reference cell and the devices under test

☒ Yes ☐ No

This is done by Enlitech

Explain why this information is not reported/not relevant.

6. Mask/aperture

Size of the mask/aperture used during testing

☒ Yes ☐ No

0.1 cm2

Explain why this information is not reported/not relevant.

Variation of the measured short-circuit current density with the mask/aperture area

☒ Yes ☐ No

Supplementary Fig. 21

Explain why this information is not reported/not relevant.

7. Performance certification

Identity of the independent certification laboratory that confirmed the photovoltaic performance

☒ Yes ☐ No

Identify the independent certification laboratory.

Explain why this information is not reported/not relevant.

A copy of any certificate(s)

☒ Yes ☐ No

Supplementary Fig. 46

Explain why this information is not reported/not relevant.

8. Statistics

Number of solar cells tested

☒ Yes ☐ No

100 cells for 5 conditions

Explain why this information is not reported/not relevant.

Statistical analysis of the device performance

☒ Yes ☐ No

Fig. 1F and Supplementary Fig. 21

Explain why this information is not reported/not relevant.

9. Long-term stability analysis

Type of analysis, bias conditions and environmental conditions

☐ Yes ☒ No

Provide a description of the type of analysis, bias conditions and environmental conditions (e.g. illumination type, temperature, atmosphere humidity, encapsulation method, preconditioning temperature, bias) for each long-term stability analysis carried out; see ref. 7 and 8 for details.

This paper does not involve stability analysis
